# Riparian Bryophytes: An Overlooked Yet Important Habitat for Aquatic Macroinvertebrates in Interior Pacific Northwest (USA) Salmonid‐Bearing Streams

**DOI:** 10.1002/ece3.72627

**Published:** 2025-12-16

**Authors:** Joshua P. Averett, Leslie M. Naylor, David E. Wooster, Amanda Hardman, Michael J. Wisdom, Bryan A. Endress

**Affiliations:** ^1^ Eastern Oregon Agricultural Research Center—Union Experiment Station Oregon State University Union Oregon USA; ^2^ Confederated Tribes of the Umatilla Indian Reservation Island City Oregon USA; ^3^ Department of Fisheries, Wildlife, and Conservation Sciences, Hermiston Agricultural Research and Extension Center Oregon State University Hermiston Oregon USA; ^4^ Malheur National Forest John Day Oregon USA; ^5^ USDA Forest Service Pacific Northwest Research Station La Grande Oregon USA

## Abstract

Restoration of riparian vegetation is an objective for recovery of threatened salmonids in the interior Pacific Northwest (PNW; USA). However, bryophytes (mosses, liverworts, and hornworts) are overlooked despite their capacity to provide stream habitat for invertebrates, an important salmonid food. The role of bryophytes as invertebrate habitat is little studied in the PNW. We investigated macroinvertebrate communities in bryophytes across three aquatic habitat types (headwaters, mid‐order streams and wetlands) in a salmonid‐bearing stream system in the Blue Mountains, northeastern Oregon (USA). We sampled paired bryophyte and non‐bryophyte benthic (streambed) substrates from 12 sites (headwaters, *n* = 3; mid‐order streams, *n* = 6; wetlands, *n* = 3) during spring, summer and fall, 2022–2023. Bryophytes, regardless of habitat, enhanced invertebrate density per square meter, by ~11 times, on average, compared to streambeds without bryophytes. Bryophytes increased the abundance of most functional groups and hosted unique invertebrate communities dominated by Chironomidae (midges) and detritivores. Bryophytes did not serve as nursery habitat; however, invertebrates were smaller (mean length ~12% shorter) in bryophytes compared to streambeds, primarily because bryophytes were dominated by smaller taxa. Non‐metric multidimensional scaling indicated that the dominant gradient in invertebrate composition was related to separation between bryophytes and streambeds (*R* ^2^ = 0.70). This gradient was driven by the dominance of midges in bryophytes compared to streambeds regardless of season or habitat type. Bryophytes harbored high densities of most functional groups of macroinvertebrates, particularly midges, a highly productive food for many stream and riparian organisms. These results suggest that bryophytes are important habitat for macroinvertebrates across most aquatic habitat types in salmonid‐bearing stream systems of the Blue Mountains. By considering bryophytes in stream‐riparian restoration and management plans, practitioners can enhance an important vegetation component and support conservation of highly productive aquatic invertebrate communities and consumers that depend on them for food.

## Graphical abstract

We investigated riparian bryophytes as habitat for aquatic invertebrates in an interior Pacific Northwest (PNW) salmonid stream system. Our results demonstrate that bryophytes are highly productive habitat for aquatic invertebrates in interior Pacific Northwest salmonid streams. By considering bryophytes in stream‐riparian restoration and management plans, practitioners can enhance an important vegetation component that can support the conservation of highly productive aquatic invertebrate communities and consumers that depend on them for food.

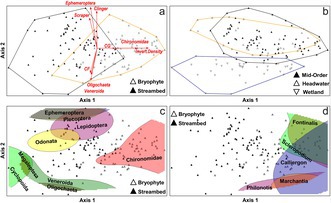

## 1 Introduction

Habitat loss and alteration is responsible for global declines in freshwater biodiversity (Dudgeon et al. 2006; Reid et al. 2019). Climate change impacts are expected to exacerbate stressors to biota in altered streams and riparian zones, further challenging the conservation of freshwater communities (Reid et al. 2019). Active habitat restoration is one of our most effective tools to increase the resiliency of degraded streams and riparian zones (Beechie et al. 2013; Van Looy et al. 2019). Declining salmonid populations associated with degraded tributary habitat for juvenile fish, have made the interior Pacific Northwest (PNW; USA) a focal point for stream and riparian restoration (Bernhardt et al. 2005; Jaeger and Scheuerell 2023). Restoration in the region focuses on improving fish production mainly through maintenance or restoration of the physical characteristics of streams (e.g., riparian vegetation cover, large wood and boulders, pool abundance and geometry; Wipfli and Baxter 2010). Traditional approaches to stream restoration in the PNW have considered fish production to be limited primarily by physical habitat rather than food. Restoration in the region is increasingly embracing the idea that physical habitat, habitat heterogeneity and food resources all interact to influence salmonid productivity in PNW streams (Kaylor et al. 2020; Wipfli and Baxter 2010). Despite huge investments for stream restoration, evaluations commonly find little difference in stream biotic communities between restored and unrestored sites (Frainer et al. 2018). One potential explanation is a failure to restore key habitat components that are important for freshwater communities (Frainer et al. 2018).

The structure and function of streams are intricately linked to riparian vegetation. Vegetation stabilizes stream banks, shades streams from solar radiation, provides large wood and organic inputs, influences aquatic and terrestrial invertebrate communities, provides habitat for aquatic and terrestrial biota, and influences stream‐channel morphology and stream‐floodplain hydrology (Allan et al. 2021). Consequently, conservation and/or recovery of riparian vegetation is a high priority for stream restoration globally (González et al. 2015). In the interior PNW, restoration of riparian vegetation, particularly woody vegetation that can shade streams, is a primary objective for recovery of threatened salmonid populations (Jones 2008; McCullough 1999; Nowack 2004; Wondzell et al. 2019). However, one potentially important riparian vegetation group−bryophytes−is overlooked (Glime 2017; Stream Bryophyte Group 1999).

Bryophytes (mosses, liverworts, and hornworts) are dominant macrophytes in mountain streams where water velocities are too high for vascular plants to establish, where impenetrable substrates (e.g., bedrock and boulders) restrict rooting by vascular plants, and in some slower creeks, seeps, and side channels. Bryophytes are vulnerable to alteration of aquatic and semiaquatic habitats, particularly flow alteration and removal of stable substrates (Glime 2020). In some parts of the world (e.g., Europe), bryophytes are recognized as indicators of stable habitat, low human alteration and disturbance, and high community stability in streams (Portela et al. 2017). Although bryophytes are expected to influence ecosystem structure and function of many streams, there are major knowledge gaps regarding their roles in such systems (Glime 2020; Stream Bryophyte Group 1999). One recognized pattern is that bryophytes often harbor disproportionately high densities of aquatic invertebrates compared to other benthic substrates (Glime 2017; Suren 1991).

Invertebrates contribute a large component to freshwater biodiversity and are important for the function of streams and riparian zones (e.g., through nutrient recycling, water purification, and processing of organic matter; Collier et al. 2016). One of the most important roles of aquatic invertebrates is as an energetic link between primary producers and higher‐level consumers (Collier et al. 2016). Aquatic macroinvertebrates are a primary food resource for juvenile salmonids and other fish (Allan et al. 2021; Armitage 1995) and provide important subsidies to many riparian predators (e.g., birds, bats, amphibians, lizards, and spiders; Armitage 1995; Baxter et al. 2005). Recent research identified that juvenile salmonids in the upper Grande Ronde River Watershed (our study region) are strongly food limited in summer (Kaylor et al. 2020). Therefore, conservation and restoration of high‐quality habitat that increases food resources for fish are important for conservation efforts in the region.

Converging evidence from temperate, arctic, and tropical streams suggests that bryophytes enhance the abundance and stability of aquatic invertebrate communities across a wide range of stream systems (Glime 2020; Lee and Hershey 2000; Munn and Brusven 1991; Muotka and Laasonen 2002; Suren 1991; Tada and Satake 1994; Wotton and Merritt 1988; Wulf and Pearson 2017). A common, although untested, hypothesis is that bryophytes are particularly good habitat for small aquatic insects (e.g., Chironomidae) and specifically serve as nurseries harboring early instars of larger invertebrates (e.g., stoneflies–Plecoptera) that move to streambeds as they mature (Glime 2017; Suren 1991; Wulf and Pearson 2017). Enhanced densities and growth rates of invertebrates in bryophytes and other macrophytes have conveyed fitness benefits to fish in Alaska (arctic grayling, *Thymallus arcticus* , Lee and Hershey 2000) and are hypothesized to improve salmonid (*Oncorhynchus* spp.) fitness in northern California (Lusardi et al. 2018). Bryophytes may have a similar role in interior PNW stream systems, particularly if they boost invertebrate productivity in summer concomitant with food limitations in the region.

Two prevailing hypotheses as to why aquatic invertebrate densities are high in bryophytes are (1) they provide a stable substrate that serves as a flow refuge (flow refuge hypothesis; Glime 2017; Suren 1992); and (2) bryophytes provide stable and concentrated food sources for some aquatic invertebrates through trapping of fine organic matter (Glime 2017). If flow refuge is the primary reason that aquatic invertebrates inhabit bryophytes, then colonization of bryophytes should decrease as one moves from faster to slow or still water habitats (Glime 2017; Suren 1992). Most research that has documented enhanced abundances of aquatic invertebrates in bryophytes has focused on flowing streams (Glime 2017; Kreuzinger‐Janik et al. 2022). Few studies have occurred in still‐water habitats (Kreuzinger‐Janik et al. 2022). Research from the tropics suggests that aquatic invertebrate abundance in bryophytes is positively associated with water velocity, that is, greatest colonization occurs during high flow periods where invertebrates apparently use bryophytes as velocity shelters (Rosa et al. 2011). Understanding the relationship between water velocity and invertebrate colonization of bryophytes is critical to understanding how broadly (both spatially and temporally) bryophyte habitats may impact invertebrate communities. If trapping organic material is the primary reason that invertebrates inhabit bryophytes, then invertebrate communities colonizing bryophytes should be strongly dominated by detritivores.

The goal of this research was to evaluate the role of riparian bryophytes as habitat for aquatic macroinvertebrates across three different aquatic habitat types (headwater streams, mid‐order streams, and wetlands) in a Blue Mountain salmonid‐bearing stream system in northeastern Oregon. Specifically, we expected: (1) higher densities of invertebrates in bryophytes compared to streambeds without bryophytes for streams, but little to no difference in wetlands (a test of the flow refuge mechanism); (2) that bryophytes would harbor unique communities of invertebrates dominated by detritivores compared to streambeds (a test of the trapping of organic material mechanism); (3) that separation between lentic (wetland) and lotic (stream) habitats would explain more variation in invertebrate community composition compared to substrate type; and (4) that bryophyte invertebrate communities would be dominated by smaller taxa including Chironomidae and early instars of larger aquatic insects (e.g., Odonata and Plecoptera), whereas larger aquatic insects would have affinities for streambed substrates.

## 2 Methods

### 2.1 Study Area

**FIGURE 1:**
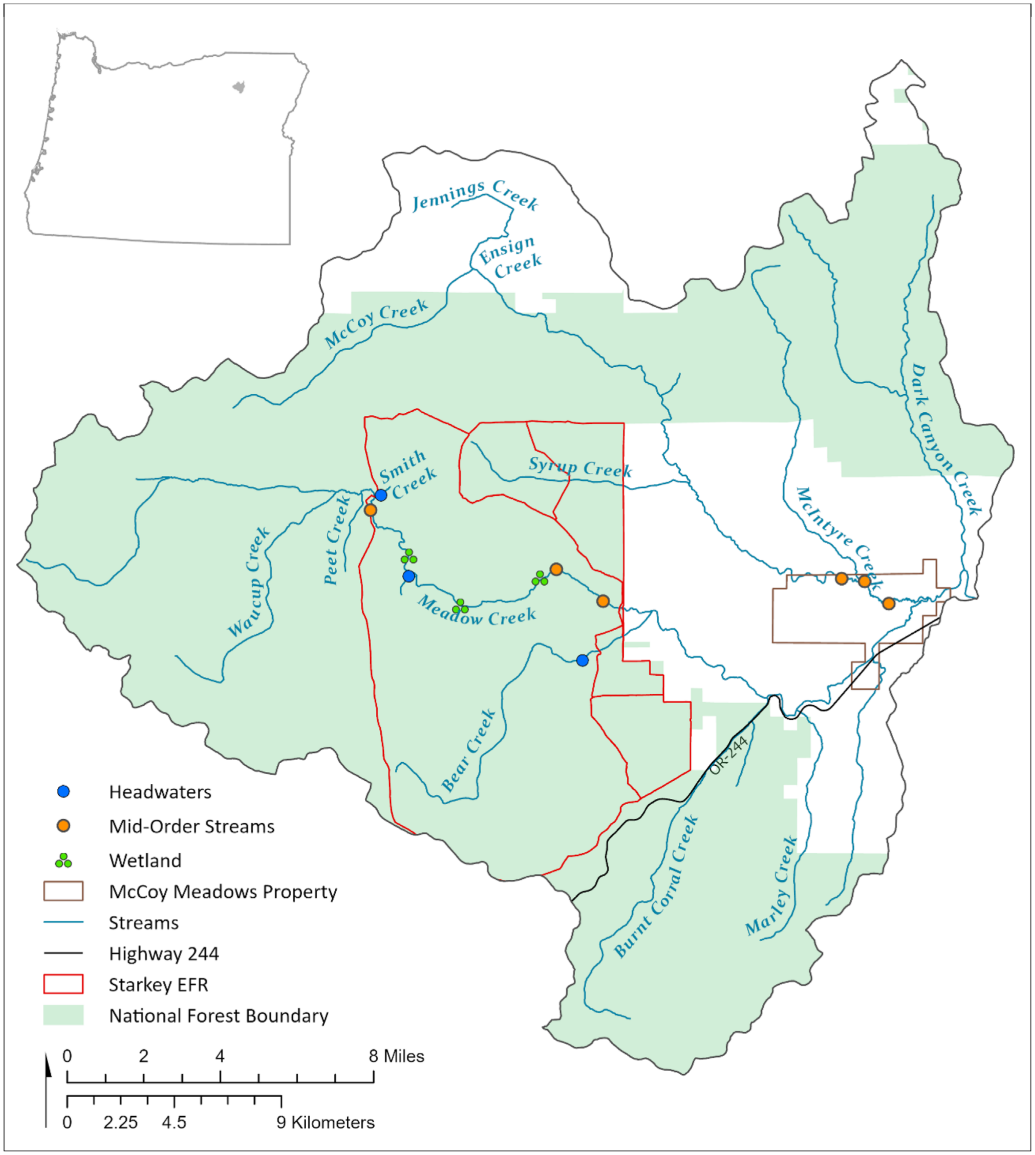
Study area (Meadow Creek Watershed). Bryophyte sampling sites are shown. Orange symbols are mid‐order stream sites, blue symbols are headwater sites, and green symbols are floodplain wetland sites. Starkey Experimental Forest and Range (Starkey EFR) is located within the Wallowa–Whitman National Forest. McCoy Meadows is property of the Confederated Tribes of the Umatilla Indian Reservation.

Meadow Creek is a mid‐order tributary of the Grande Ronde River located in the Blue Mountains in the Columbia River Basin (Figure 1). We sampled aquatic macroinvertebrates (length ≥ 500 μm) from bryophytes and streambeds in 12 sites across three water body types (headwaters, mid‐order streams, wetlands) in the Meadow Creek study area (Figure 1). Nine sites were located within the U.S. Forest Service Starkey Experimental Forest and Range (SEFR) and three sites were within the Confederated Tribes of the Umatilla Indian Reservation (CTUIR) McCoy Meadows Conservation property (Figure 1). Elevations in our study area ranged between ~1120 and 1500 m, air temperatures ranged from −1.5°C in December to 18°C in August (1990–2020), and annual precipitation averaged 569 mm (1990–2020; PRISM Climate Group 2024). Precipitation in SEFR occurs primarily as snow between November and April and the climate is semi‐arid with a predictable summer drought. Upland vegetation in our study area is primarily dry mixed conifer forest with shallow soil inclusions dominated by bunchgrasses and shrubs. Forests are dominated by Douglas fir ( *Pseudotsuga menziesii* ) with either ponderosa pine ( *Pinus ponderosa* , south‐facing slopes) or grand fir ( *Abies grandis* , north‐facing slopes). Lodgepole pine ( *Pinus contorta* ) and Engelmann spruce ( *Picea engelmannii* ) are common along headwater streams and cold‐air drainages. Understory riparian (vascular) vegetation in the study area is dominated by graminoids including Northwest Territory sedge ( *Carex utriculata* ) and wooly sedge ( *Carex pellita* ), panicled bulrush ( *Scirpus microcarpus* ), arctic rush ( *Juncus arcticus* ), and creeping bentgrass ( *Agrostis stolonifera* ). Narrowleaf willow ( *Salix exigua* ) shrubs and thinleaf alder ( *Alnus incana* ssp. *tenuifolia* ) trees are common overstory species.

Bryophytes readily colonize headwater stream channels in our study area (Averett 2025) but because past human activities have greatly reduced the abundance of stable in‐stream substrates, bryophytes are primarily constrained along stream margins in mid‐order streams (Averett 2025). Dominant bryophytes along Meadow and McCoy Creeks included the mosses *Calliergon* spp., *Philonotis fontana* , and *Brachythecium* spp. and liverworts (*Marchantia* spp). Localized areas with stable in‐stream substrate (e.g., logs and boulders) support *Fontinalis antipyretica* and *Scleropodium obtusifolium* (aquatic mosses) in both Meadow and McCoy Creeks. Bryophytes are most abundant in headwater reaches of our study area (average cover 34%–68% of the bank‐full channel; Averett 2025) where *Fontinalis* and *Scleropodium* are dominant. Bryophytes are common in floodplain wetlands (average cover ~15%–38%; Averett 2025) along Meadow Creek. The three wetlands sampled in this study all originate from springs located near the toe slope of the valley bottom along Meadow Creek (Averett 2025). Dominant bryophytes in two wetlands fed from cold groundwater (Alder Springs and Larch Springs) included *Calliergon*, *Plagiomnium* spp., liverworts, *Rhytidiadelphus squarrosus* and *Pleurozium schreberi* . The dominant bryophyte in the third wetland, Warm Springs (geothermal spring; temp ~25.6°C) was *Philonotis fontana* (Averett 2025).

Stream flows in our study area are characteristic of a snowmelt hydrograph, with peak flows typically occurring in late winter and early spring (e.g., March and April). Baseflow conditions occur during summer through early fall. Meadow Creek and McCoy Creek have ~1%–2% gradients (US Forest Service, La Grande Ranger District 2001) with annual average discharges of ~0.25–0.85 cms (10–30 cfs), peak discharges of ~8.5–14 cms (300 to > 500 cfs) and summer discharges of < 0.05 cms (2 cfs; Oregon Water Resources Department gage 13318060). The three headwater streams (Bear Creek, Cougar Creek, and Smith Creek) are all spring fed with annual average discharges < 10% of Meadow Creek (Wood 2017). Stream gradients for Bear and Smith Creeks are ~2%–4% and ~4%–6% for Cougar Creek (US Forest Service, La Grande Ranger District 2001).

### 2.2 Sampling

We conducted a two‐year (2022–2023) study across 12 sites (three headwaters, three wetlands, and six mid‐order stream sites), collecting aquatic macroinvertebrates from 72 paired bryophyte and streambed substrate replicates (total sample size = 144; Figure 1). We sampled 36 paired replicates from mid‐order stream sites and 18 paired replicates each from headwater and wetland sites. Sampling occurred during spring, summer, and fall bouts each year. Study reaches varied in length dependent upon the amount of bryophyte cover and ranged from 200 to 400 m. Sampling in spring was started when stream flows decreased to safe levels for wading (May or June). Wetland sites varied in size from ~0.25 to 1 ha. Each invertebrate sample was composed of six subsamples composited into a single sample (streambed composite sample area = 0.56 m^2^; bryophyte composite sample area = 0.14 m^2^). Subsamples were spread out (along each stream reach and across each wetland) to obtain good site coverage during each sampling bout.

Bryophytes were collected (0.023 m^2^; 15.24 cm by 15.24 cm for each subsample) using hand grabs (Glime 2017; Suren 1991; Wulf and Pearson 2017) placed into a D‐frame kick‐net (500 μm mesh) located immediately downstream of the bryophyte section. If vascular vegetation (e.g., sedges) was growing intermingled within bryophytes, pruning shears were used to cut vascular plant material above the bryophyte surface prior to bryophyte removal. The bryophyte sample was cut away from adjacent bryophyte material with shears prior to removal. If the collected bryophyte sample was attached to rock surfaces, remaining bryophyte material was scraped from the rock surface into the net using a flexible 5‐in. (12.7 cm) metal drywall knife (semi‐circular joint knife). After removal, all remaining vascular plant material (e.g., leaves and roots) was manually removed from the bryophyte samples and discarded. Bryophyte samples were transferred into 1 L sample jars containing 95% ethanol in the field. Streambed samples were taken from the closest riffle habitat, or glide if riffles were not present, upstream of each bryophyte sample (≥ 2 m upstream of each paired bryophyte sample). At each streambed site, benthic macroinvertebrates were sampled using a kick‐net (500 μm; 0.093 m^2^ area sampled for each subsample). All substrate items larger than ~7 cm were manually scrubbed with a stiff brush in front of the net followed by a 30‐s kick‐sample. For kick samples where bedrock was encountered within 5 cm below the streambed surface, the sample area was scrubbed with a stiff brush for 30 s in front of the net. For streambed subsamples from wetlands, benthic material to a ~3 cm depth was collected into the net. Substrate comparisons were made using a standardized 1 m^2^ sampling area.

Streambed samples were transferred into 1 L sample jars containing 95% ethanol in the field. At each subsample (bryophyte and streambed), we measured water depth (cm), water velocity (m/s), and canopy openness (average of four densiometer measurements in the cardinal directions), and estimated bryophyte composition (% cover by genus of composite samples). Invertebrate collections were processed in the lab by subsampling (Caton 1991; OWEB 1999). Collections were evenly spread on a gridded tray (30 cells; cell dimensions = 6 × 6 cm). Cells were subsampled at random (minimum of four cells), completely sorted using a stereo dissecting scope within selected search squares until > 300 individuals were counted (OWEB 1999). If 300 individuals were not counted within the first four cells, then additional cells were selected until at least 300 were counted; all cells subsampled were completely sorted even if the counts far exceeded 300 to allow for estimation of invertebrate abundances for the entire sample. Sorted invertebrates were sent to the National Aquatic Monitoring Center (NAMC; Utah State University) for taxon identification. Invertebrates were identified to taxon following the Southwest Association of Freshwater Invertebrate Taxonomist (SAFIT‐2a) standard taxonomic effort (Richards and Rogers 2011). Non‐insects were identified to order, mature insects were identified to genus and species (immature individuals identified to coarser taxonomic levels) except for Chironomidae (non‐biting midges) which were identified to subfamily. Taxon abundances were estimated for each sample by dividing the number of subsampled individuals by the proportion of the entire sample sorted and then standardized to 1 m^2^ based on the sample area for each given substrate.

Body length measurements were made by taxon for samples from summer 2022, fall 2022, spring 2023 and from a random selection of samples (~50%) from summer and fall of 2023. For each sample, body length measurements, to the nearest 0.2 mm, were made for 25 (or all individuals if *n* ≤ 25) randomly selected individuals from each taxon.

## 3 Data Analysis

### 3.1 Invertebrate Densities and Diversity

All analyses were performed using R version 4.3.2 (R Core Team 2023) if not otherwise noted. We calculated richness (*S*), Shannon's diversity index (*H*′), Simpson's diversity index for infinite population (*D*), Whitaker's beta diversity (*β* ^W^), beta half changes (*β* ^D^), and rank frequency and abundances for invertebrates (PC‐ORD version 7.0; McCune and Mefford 2015). Bootstrapping was used (10,000 replicates) to generate means and 95% confidence intervals (CI; percentile method; Davison and Hinkley 1997) for the paired differences (bryophyte density–streambed density) in invertebrate density between bryophytes and streambeds. Means and CIs were calculated for all invertebrates combined and additionally for dominant invertebrate orders. Paired density ratios (invertebrate density in bryophytes/invertebrate density in the streambed at a given site on the same date) were also calculated and plotted to allow for efficient visualization and coarse comparisons of density differences between bryophytes and non‐bryophyte streambeds; this was done to relativize the density data because of high variation in invertebrate densities across sampling bouts and habitat types.

### 3.2 Community Gradients

Non‐metric multidimensional scaling (NMS; McCune and Mefford 2015) using a Sørenson distance measure, Kruskal's strategy 1 for penalization of ties, and the slow and thorough autopilot setting was used to extract the dominant gradients of invertebrate community composition from our dataset. NMS was performed from a random starting configuration and a maximum of 500 iterations. Rare invertebrate orders occurring in less than 5% of plots (7 out of 144) were removed from the species matrix prior to analysis to reduce noise and enhance any signal related to dominant invertebrate orders' relationships to community dissimilarity between plots. NMS was performed on a final species matrix of 144 sample units by 24 invertebrate orders. We chose to investigate community gradients on the order level because we were interested, from a food‐web perspective, in understanding how substrate type (bryophytes vs. streambeds) influenced broad community patterns for different functional groups of invertebrates. Further analyses (described below) were used to delineate finer‐level taxon relationships with the ordination space and specific substrates. Joint plots were used to evaluate linear correlations between important variables (e.g., taxon density and relative abundance, traits, and abiotic factors) with the ordination space (McCune and Grace 2002). Classification of invertebrate traits was provided by the National Aquatic Monitoring Center. Traits included five functional feeding groups (collector‐gatherers, collector‐filterers, scrapers, shredders, predators) and six behavioral groups (clinger, climber, skater, sprawler, burrower, swimmer; see Cummins et al. 2019 for more information regarding feeding and habit (behavioral) groups). Reliance only on linear relationships can result in overlooking variables that are strongly associated with ordination axes. Therefore, we also evaluated nonlinear responses of biotic and abiotic factors with the ordination space using non‐parametric multiplicative regression (NPMR; McCune 2006). NPMR is a multiplicative kernel smoother which automatically models interactions between the response and predictors and has built‐in over‐fitting protection. Hilltop plots were used to overlay the maximum 15% range value of NPMR responses for multiple invertebrate and bryophyte taxa onto the ordination space; this allowed us to evaluate nonlinear relationships between abundances of multiple taxa (simultaneously) and the ordination space (Nelson et al. 2015).

We performed another NMS ordination using a log transformed species abundance matrix to evaluate how influential dominant taxa were in defining the ordination structure. Species abundances were log transformed to give subdominant taxa more weight in defining the ordination structure while maintaining monotonicity with the raw data (McCune and Grace 2002). We used a generalized log transformation, *b* = log(*x* + *x*_min_) − log(*x*_min_), where *x*_min_ is the smallest nonzero value in the matrix, because the smallest non‐zero numbers were much less than one.

We tested whether invertebrate communities differed between bryophytes and streambeds as well as across aquatic habitats using multi‐response permutation procedure (MRPP; Mielke and Berry 2001) with a Sørenson distance measure. MRPP calculates an *A*‐statistic, a measure of within‐group homogeneity. An *A*‐statistic value = 1 indicates that all components of a group are identical, that is, differences between groups are as large as possible (given the dataset); and an *A*‐statistic value = 0 indicates within‐group homogeneity meets random expectation (McCune and Grace 2002). MRPP was performed for the same species matrix used for NMS. Indicator species analysis (ISA; McCune and Mefford 2015) was used to identify invertebrates that had tendencies towards either bryophyte or streambed habitats. ISA was performed using a finer level (SAFIT‐2a) classification of taxa resulting in a species matrix of 144 plots by 335 taxa (see Averett 2025 for taxon list). ISA uses both relative abundance and frequency information to calculate indicator values (IV) that represent the strength of tendencies for each taxon to occur in a priori groups (McCune and Grace 2002).

### 3.3 Effect of Substrate Type on Aquatic Insect Size

Mixed‐effects models (lme4 package; Bates et al. 2015) were used to test the hypothesis of no effect of substrate type (bryophyte vs streambed) on invertebrate size (length) after accounting for confounding variables. We performed separate analyses for dominant invertebrate orders, Coleoptera, Diptera, Ephemeroptera, Odonata, Plecoptera, and Trichoptera. We limited analyses to dominant orders to ensure large enough sample sizes for reliable inference. We chose to evaluate orders separately because we were particularly interested in the potential of bryophytes serving as nurseries for some invertebrates; combining orders that varied considerably in size (Odonata and Diptera) may have masked any signal related to intra‐taxon size differences between substrates. For all models, site was included as a random effect and length (mm) was the response variable. Substrate, aquatic habitat, season, and year were fixed effects. We ran separate models where invertebrate family was also included as a fixed effect to determine if substrate effects on invertebrate size were dominated by inter‐taxa or intra‐taxon size segregation between substrates.

## 4 Results

### 4.1 Invertebrate Densities and Diversity

**FIGURE 2:**
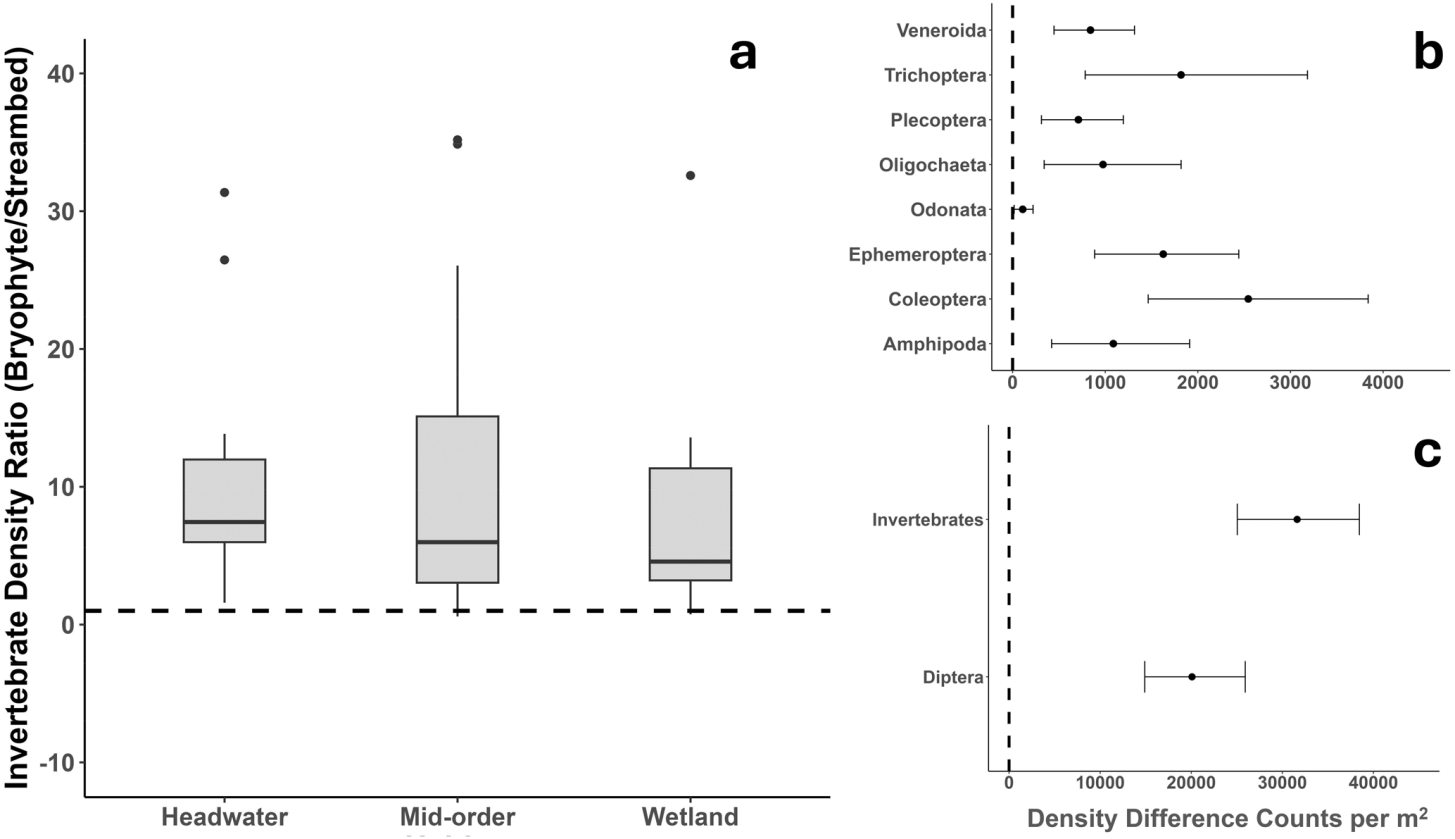
(a) Boxplots of invertebrate density (paired) ratios (invertebrate counts per m^2^ in bryophytes/invertebrate counts per m^2^ in the streambed for a given date at the same site; left panel). A *Y*‐value of 10 means that invertebrate density in bryophytes for that site, on a given date, was 10 times greater compared to invertebrate density in the streambed for that same site, on the same date. (b, c) Bootstrapped means and 95% CIs for differences in invertebrate density between substrates (bryophyte density–streambed density) for selected invertebrate orders (right panel). (c) Diptera are shown separately because they dominated invertebrate densities in both substrates and obstruct visualization of patterns of other taxa when plotted on the same graph. One outlier point (*Y*‐value = 145.0) for headwater streams is not shown in the boxplot because including that point makes it difficult to see information contained in boxplots.

**FIGURE 3:**
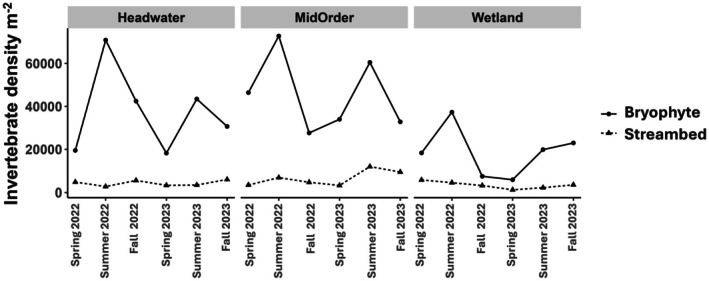
Line graphs comparing mean invertebrate densities per square meter over time between bryophyte and streambed substrates across aquatic habitat types in the Meadow Creek study area, northeastern Oregon. A total of 12 sites were sampled (three headwaters, three wetlands, and six mid‐order stream sites) during spring, summer, and fall in 2022 and 2023. Each point for headwaters and wetlands in the graph represents an average density for three sites during a given sampling bout; each point for mid‐order streams is an average density of six sites during a given sampling bout.

Average invertebrate densities per square meter (across all sites and sampling bouts) were about seven times greater in bryophytes (36,893 m^−2^) compared to streambeds (5248 m^−2^). However, after accounting for the dependency of samples taken from the same site on the same date (paired samples), we estimated that invertebrate densities per square meter were 11.3 times higher (on average) in bryophytes compared to streambeds (paired difference = 31,548 m^−2^; 95% CI = 25,146 m^−2^, 38,451 m^−2^; Figure 2). Bryophytes enhanced macroinvertebrate densities most in headwater streams (mean = 17.7 times greater) and least in wetlands (mean = 7.9 times greater; Figure 2). Variation in overall invertebrate densities and paired differences between substrates were greatest in summer for all aquatic habitats (Figure 3). Bryophytes enhanced invertebrate densities most in summer (17.1 times greater; 95% CI = 9.1, 30.1) followed by spring (11.1 times greater; 95% CI = 7.3, 15.3) and least in the fall (5.7 times greater; 95% CI = 4.3, 7.2). Densities of most invertebrate orders were enhanced in bryophytes compared to streambeds, particularly for Diptera (e.g., Chironomidae), which made up ~64% of the average difference in densities between bryophytes and streambeds (Figure 2). Invertebrate densities were consistently greatest in bryophytes during the summer for all habitats (~57,000 m^−2^ in headwaters, ~67,000 m^−2^ for mid‐order streams and ~30,000 m^−2^ for wetlands) coincident with the highest productivity and low stream discharge (Figure 3).

Invertebrate richness and diversity were higher in streambeds (*S* = 33.0, 95% CI = 31.1, 35.0, *H*′ = 2.36, 95% CI = 2.23–2.48) compared to bryophytes (*S* = 27.5, 95% CI = 25.7–29.4, *H*′ = 1.97, 95% CI = 1.85–2.09) for stream habitats (Table 1). In contrast, wetland invertebrate richness and diversity were greater in bryophytes (*S* = 24.3, 95% CI = 22.5–26.3, *H*′ = 2.24, 95% CI = 2.14–2.35) compared to streambeds (*S* = 19.5, 95% CI = 17.3–21.7, *H*′ = 1.89, 95% CI = 1.69–2.09; Table 1). Beta diversity (*β* ^w^) and beta half changes (*β* ^D^) were greater for streambeds in wetlands and similar for bryophytes and streambed invertebrate communities for streams and all aquatic habitats combined (Bryophytes, *β* ^w^ = 8.6, *β* ^D^ = 2.0; Streambeds, *β* ^w^ = 8.5, *β* ^D^ = 2.3; Table 1). Overall, beta diversity for invertebrate communities increased when data from both substrates (bryophytes and streambeds) were combined (streams and wetlands; *β* ^w^ = 11.1, *β* ^D^ = 2.5).

**TABLE 1:** Diversity statistics for invertebrate communities sampled from bryophyte and streambed substrates across aquatic habitats (headwater streams, mid‐order streams and wetlands) in a Blue Mountain stream system, northeastern Oregon. 95% CIs are shown parenthetically.

	*S*	*E*	*H*′	*D*	*β*_w_	*β*_D_
Streams (*n* = 54)						
Bryophyte	27.5 (25.7, 29.4)	0.60 (0.56, 0.63)	1.97 (1.85, 2.09)	0.74 (0.70, 0.77)	7.2	1.9
Streambed	33.0 (31.1, 35.0)	0.68 (0.64, 0.71)	2.36 (2.23, 2.48)	0.81 (0.77, 0.84)	6.5	2.0
Wetlands (*n* = 18)						
Bryophyte	24.3 (22.5, 26.3)	0.71 (0.68, 0.73)	2.24 (2.14, 2.35)	0.83 (0.81, 0.85)	3.9	1.5
Streambed	19.5 (17.3, 21.7)	0.65 (0.58, 0.71)	1.89 (1.69, 2.09)	0.73 (0.67, 0.79)	4.7	1.9
All habitats (*n* = 72)						
Bryophyte	26.7 (25.2, 28.2)	0.63 (0.60, 0.65)	2.04 (1.94, 2.13)	0.76 (0.73, 0.79)	8.6	2.0
Streambed	29.6 (27.6, 31.7)	0.67 (0.64, 0.70)	2.24 (2.12, 2.36)	0.79 (0.76, 0.82)	8.5	2.3

*Note:* *S* = richness; *E* = evenness; *H*′/ln(S); *H*′ = Shannon's diversity index; *D* = Simpson's diversity index; *β*_w_ = Whitaker's beta; *β*_D_ = dissimilarity beta (half changes).

### 4.2 Community Gradients

**FIGURE 4:**
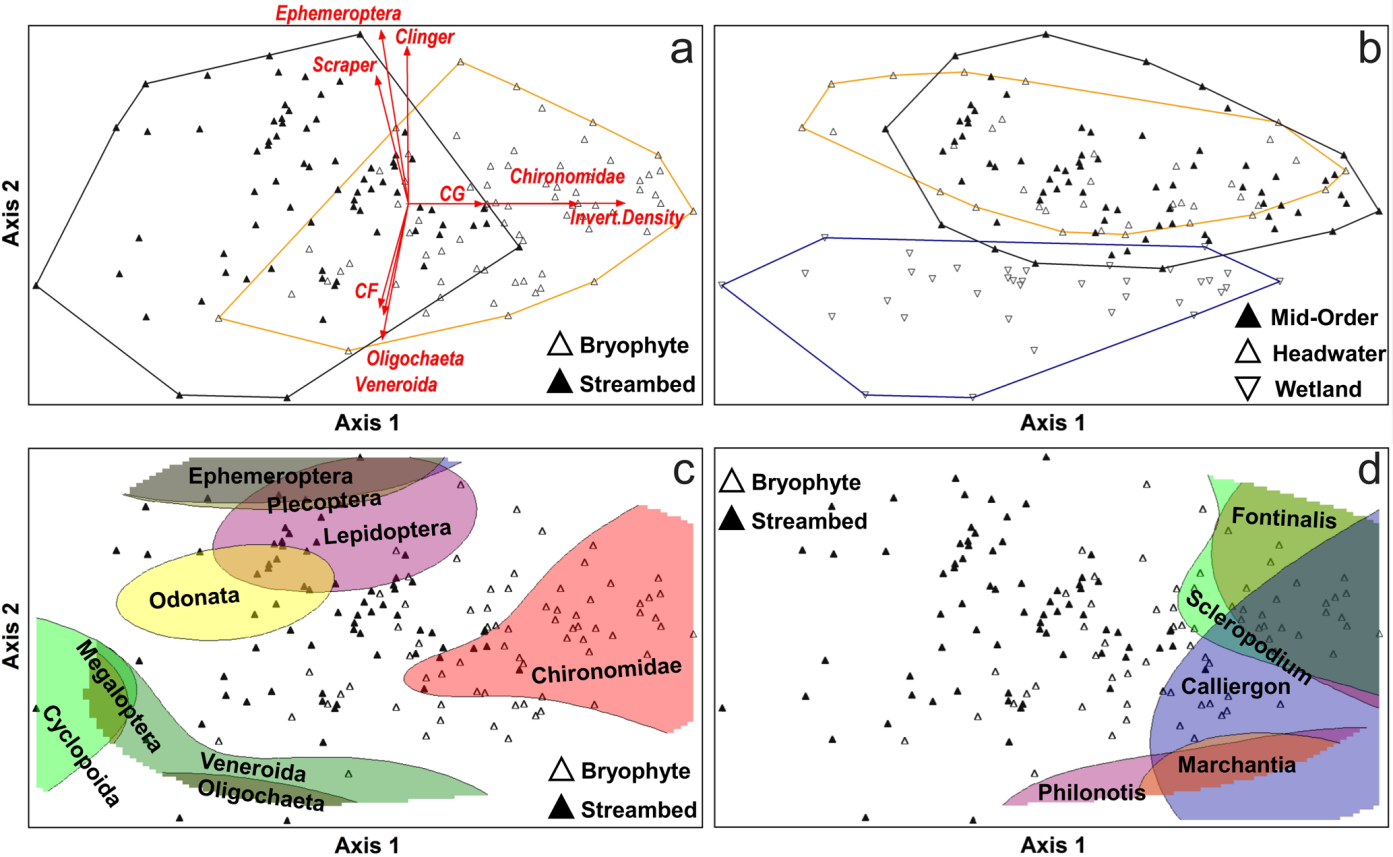
Non‐metric multidimensional scaling (NMS) ordination of sample units in macroinvertebrate community space (2‐dimensional solution explaining 82% of variation in the distance matrix; final stress = 10.9). Sample units represent *n* = 72 paired bryophyte and streambed substrate samples collected from three headwaters, three wetlands, and six mid‐order stream sites in the Meadow Creek study area (northeastern Oregon) during spring, summer, and fall of 2022 and 2023. Dominant community gradients were Axis 1 (*R* ^2^ = 0.70; representing separation between bryophyte and streambed substrates) and Axis 2 (*R* ^2^ = 0.12; separating lotic and lentic habitats). Convex hulls show maximum area of sample units for groupings based on substrate (panel a) and aquatic habitat types (panel b). (a) Joint plot; vectors show direction and strength (length of vectors) of linear correlations between relative abundances of invertebrate traits and taxa with ordination scores. CF is collector‐filterer, CG is collector‐gatherer, invert. density is total density per square meter of invertebrates. (c, d) Hilltop plots show non‐linear regression responses (maximum 15% range of values, i.e., peak abundance in ordination space) of multiple taxa overlaid on the ordination space: (c) relative abundance of macroinvertebrate groups, and (d) bryophyte abundance (% cover) by genus.

A two‐dimensional ordination (Figure 4; final stress = 10.9; randomization test; *p* = 0.004) explained 82% of the variation in the distance matrix. The two substrate types, bryophytes versus streambeds, separated clearly along Axis 1 (Figure 4). Invertebrate composition differed between bryophytes and streambeds (MRPP; *A* = 0.11; *p* < 0.001) and invertebrate community heterogeneity across aquatic habitat types was similar for bryophytes (*A* = 0.09; *p* < 0.001) and streambeds without bryophytes (*A* = 0.10; *p* < 0.001).

#### 4.2.1 Primary Community Gradient

Axis 1 explained most of the variation (70%) in the distance matrix and represented a clear gradient separating bryophytes (on the right) from streambeds (on the left; Figure 4). Factors with strong positive correlations with Axis 1 (associated with bryophytes), were total invertebrate density (*r* = 0.81), aquatic insects (*r* = 0.79), collector‐gatherers (*r* = 0.77), Chironomidae (*r* = 0.72), dominance by one family (proportion of sample from the most dominant family in that sample; *r* = 0.53), and *Fontinalis* cover (*r* = 0.53). Absolute abundances (densities) for most invertebrate orders (27 out of 35; ~77%), functional feeding, and behavioral groups were positively associated with bryophytes (Table 2). Any negative relationships between Axis 1 and absolute abundances of invertebrates were relatively weak (ranging from −0.014 for Megaloptera to −0.106 for Nematomorpha; Figure 4; Table 2). Relative abundance of Diptera, particularly Chironomidae, increased strongly on Axis 1 corresponding to the presence of bryophytes (Figure 4). Relative abundances of Ephemeroptera and Plecoptera were highest at the center of Axis 1, suggesting low faithfulness towards either substrate type (Figure 4). Cyclopoida and Megaloptera relative abundances were highest at low Axis 1 scores, corresponding to streambeds (Figure 4).

**TABLE 2:** Correlation coefficients (*r*) for linear relationships between abiotic/biotic variables and ordination axes along with *R* ^2^ values for NPMR models.

	Axis1 *R* ^2^	Axis2 *R* ^2^	NMPR *xR* ^2^
Abiotic variables			
Canopy openness (%)	0.091	−0.048	−0.015
Discharge (cms)	0.670	0.071	−0.009
Velocity (m/s)	−0.073	0.373	0.142
Bryophyte cover (%)			
*Fontinalis*	0.526	0.118	0.353
*Scleropodium*	0.356	0.108	0.127
*Calliergon*	0.274	−0.085	0.032
*Marchantia*	0.119	−0.257	0.105
*Philonotis*	0.109	−0.303	0.119
*Plagiomnium*	−0.026	−0.283	0.036
*Brachythecium*	−0.170	−0.082	0.007
Invertebrate traits			
Family dominance	0.530	−0.082	0.372
Collector‐gatherer	0.477	−0.022	0.302
Intolerant (density)	0.459	0.256	0.342
Tolerant (density)	0.316	−0.024	0.095
Shredder	0.120	0.307	0.090
Sprawler	0.027	0.111	0.135
Climber	−0.011	0.225	0.363
Clinger	−0.084	0.791	0.698
Skater	−0.092	0.136	0.002
Burrower	−0.121	−0.428	0.380
Swimmer	−0.179	0.473	0.272
Predator	−0.262	−0.286	0.226
Collector‐filterer	−0.295	−0.560	0.343
Scraper	−0.311	0.627	0.517
Invertebrate taxa			
Invertebrate (density)	0.812	0.048	0.746
Insecta (density)	0.786	0.108	0.733
Chironomidae	0.571	−0.121	0.465
Odonata	−0.095	0.123	0.029
Lepidoptera	−0.137	0.261	0.097
Plecoptera	−0.163	0.545	0.278
Cyclopoida	−0.232	−0.085	0.034
Oligochaeta	−0.274	−0.584	0.387
Ephemeroptera	−0.290	0.720	0.622
Veneroida	−0.281	−0.642	0.453
Megaloptera	−0.338	−0.141	0.131
Trichoptera	0.136	0.399	0.157
Coleoptera	−0.109	0.381	0.115

*Note:* Metrics for variables are relative abundance unless absolute abundance (density, i.e., absolute counts per meter squared) is indicated. For discharge, cms is cubic meters per second for aquatic habitats.

#### 4.2.2 Secondary Community Gradient

Axis 2 explained 12% of the variation in the distance matrix and was related to the separation of streams from wetlands (Figure 4). Axis 2 was most positively related to streams and relative abundances of Ephemeroptera (*r* = 0.72), clingers (*r* = 0.79), scrapers (*r* = 0.63), Plecoptera (*r* = 0.55) and swimmers (*r* = 0.47; Figure 4 and Table 2). Axis 2 was most negatively related to wetlands and relative abundances of Veneroida (*r* = −0.64), collector‐filterers (*r* = −0.56), and Oligochaeta (*r* = −0.55; Figure 4; Table 2). Water velocity (*r* = 0.37) was positively associated with Axis 2 (Table 2). Moss composition varied along Axis 2, with aquatic mosses associated with flowing waters (e.g., *Fontinalis* and *Scleropodium*) located high to mid‐way along Axis 2 (Figure 4). *Calliergon*, a moss associated with slower flowing waters along stream margins, was most abundant mid‐way along Axis 2, and bryophytes associated with wetlands (e.g., *Philonotis* and *Marchantia* spp.) were most abundant low along Axis 2 (Figure 4).

#### 4.2.3 Nonlinear Relationships Between Invertebrates and Ordination Axes

Hilltop plots, displaying the top 15% of relative abundance for specific taxa showed non‐linear relationships between the abundances of dominant aquatic invertebrates, riparian bryophyte cover, and ordination axes (Figure 4). Chironomidae was clearly associated with bryophyte substrates; in contrast Megaloptera and Cyclopoida had peak relative abundances in streambeds (Figure 4; Tables 2 and 3). Ephemeroptera and Plecoptera relative abundances were mostly independent of substrate type and increased with water velocity (Figure 4; Table 2). Veneroida relative abundance was highest in wetlands but mostly independent of substrate type (Figure 4; Table 3). Lepidoptera, Odonata, and Oligochaeta all had the highest relative abundances in streambeds but separated along Axis 2, with Odonata being associated with intermediate to slow water velocities and Oligochaeta being most dominant in wetlands (Figure 4; Table 3).

**TABLE 3:** Contribution of dominant invertebrate groups for paired bryophyte and streambed substrates collected during spring, summer and fall of 2022 and 2023 in a Blue Mountain (northeastern Oregon) stream system.

	Bryophyte	%	Streambed	%
Headwater stream (*n* = 18)	Chironomidae	60 (61)	Chironomidae	44 (35)
	Trichoptera	13 (3)	Ephemeroptera	23 (13)
	Ephemeroptera	8 (7)	Coleoptera	11 (3)
	Diptera (other)	7 (4)	Plecoptera	9 (5)
	Coleoptera	6 (6)	Diptera (other)	5 (7)
	Plecoptera	5 (3)	Trichoptera	4 (3)
			Oligochaeta	1 (1)
	Total	99%		97%
	Mean density	37,540 m^−2^		4317 m^−2^
	SD	26,911 m^−2^	SD	3147 m^−2^
Mid‐order stream (*n* = 36)	Chironomidae	49 (50)	Chironomidae	40 (37)
	Coleoptera	12 (7)	Ephemeroptera	26 (23)
	Ephemeroptera	11 (7)	Coleoptera	10 (8)
	Diptera (other)	6 (7)	Plecoptera	5 (4)
	Oligochaeta	5 (1)	Trichoptera	5 (4)
	Amphipoda	5 (< 1)	Diptera (other)	3 (3)
	Trichoptera	4 (2)	Odonata	2 (2)
	Plecoptera	2 (1)	Trombidiformes	2 (1)
	Veneroida	2 (< 1)	Amphipoda	2 (0)
	Total	96%		95%
	Mean density	45,696 m^−2^		6621 m^−2^
	SD	32,836 m^−2^		4495 m^−2^
Wetland (*n* = 18)	Chironomidae	34 (32)	Chironomidae	32 (31)
	Diptera (other)	23 (24)	Oligochaeta	23 (13)
	Veneroida	19 (17)	Veneroida	16 (13)
	Oligochaeta	12 (7)	Diptera (other)	16 (23)
	Coleoptera	4 (3)	Coleoptera	5 (2)
	Araneae	2 (1)	Megaloptera	1 (< 1)
	Trombidiformes	2 (< 1)	Eucopepoda	1 (0)
			Ephemeroptera	1 (0)
	Total	96%		95%
	Mean density	18,642 m^−2^		3433 m^−2^
	SD	13,488 m^−2^		2586 m^−2^

*Note:* Mean and median (indicated parenthetically) percentage of total counts (sample‐level) are shown for dominant taxa. Dominant bryophytes included: (headwaters) *Scleropodium obtusifolium* and *Fontinalis antipyretica* (mid‐order streams) *Calliergon* spp., *F. antipyretica* , *Brachythecium* spp., and *S. obtusifolium* , and (wetlands) *Philonotis fontana* , *Marchantia* spp., and *Plagiomnium* spp. in wetlands.

#### 4.2.4 Indicator Species Analysis

ISA identified 41 indicator invertebrate taxa for bryophytes and 18 for streambeds (Table 4). The strongest indicator taxa, by far, had affinities for bryophytes and included several sub‐families of non‐biting (Chironomidae) and biting (*Probezzia*) midges. Overall, Chironomidae showed a strong affinity for bryophytes (IV = 60.0; Table 4). However, two sub‐families of Chironomidae, Orthocladiinae (IV = 94.9) and Tanypodinae (IV = 82.9), had extremely strong indicator values suggesting that these taxa, particularly Orthocladiinae, were near perfect indicators of bryophytes in this system (Table 4). The strongest streambed indicators included stonefly taxa including *Calineuria californica* (western stonefly; IV = 51.9) and *Sweltsa* (genus of green stoneflies; IV = 36.9), as well as mayflies in the family Heptageniidae (flatheaded mayflies; IV = 46.2) and the beetle genus *Psephenus* (water penny beetles; IV = 35.7; Table 4). Separation of taxa from the same order across substrates was common. Those taxa often differed in traits that were associated with ordination axes. For example, Ephemeroptera that were collector‐gatherers (e.g., Lepthophlebiidae and Paraleptophlebia) had tendencies towards bryophytes whereas Ephemeroptera that were scrapers (Heptageniidae) were more dominant in streambeds (Table 4). Predators and scrapers were highly represented (~67%; 10 out of top 15) as indicators of streambeds. Collector‐gatherers and shredders (~53%; 8 out of top 15) were highly represented as indicators of bryophytes (Table 4).

**TABLE 4:** Select indicator invertebrate taxa for bryophytes and streambed substrates showing taxon frequency (proportion of plots where present) and abundance (mean density per square meter) for each substrate, indicator value (IV), and associated *p*‐value for each group. FFG is functional feeding group (CG, collector‐gatherer; CF, collector‐filterer; PR, predator; SC, scraper; SH, shredder; UN, unknown).

Taxa	Order	FFG	BFG	Frequency	Abundance	IV	*p*
Bryophyte (*n* = 72)							
Orthocladiinae	Diptera	CG	UN	1.00	13,214	94.9	< 0.001
Tanypodinae	Diptera	PR	UN	0.96	1749	82.9	< 0.001
Chironominae	Diptera	CG	UN	0.96	5281	73.4	< 0.001
*Probezzia*	Diptera	PR	Burrower	0.67	603	63.7	< 0.001
Pisidium	Veneroida	CF	UN	0.51	927	44.4	0.029
*Hydraena*	Coleoptera	UN	Clinger	0.40	292	40.0	< 0.001
*Dasyhelea*	Diptera	CG	Sprawler	0.47	415	39.9	0.002
Araneae	Araneae	UN	UN	0.39	91	38.2	< 0.001
Collembola	Collembola (Class)	CG	UN	0.39	59	37.9	< 0.001
*Lepidostoma*	Trichoptera	SH	Clinger	0.46	368	36.8	0.039
*Hyalella*	Amphipoda	CG	UN	0.36	1125	34.7	0.002
*Dicranota*	Diptera	PR	Burrower	0.35	68	33.7	< 0.001
*Tipula*	Diptera	SH	Burrower	0.36	67	33.5	< 0.001
Ceratopogonidae	Diptera	PR	UN	0.35	110	30.2	< 0.001
*Paraleptophelebia*	Ephemeroptera	CG	Swimmer	0.33	639	29.1	0.058
*Micrasema*	Trichoptera	SH	Clinger	0.29	1112	28.6	0.006
Leptophlebiidae	Ephemeroptera	CG	UN	0.38	180	26.1	0.097
*Isoperla*	Plecoptera	PR	Clinger	0.24	52	22.1	0.001
*Pteronarcys*	Plecoptera	SH	Clinger	0.19	40	19.3	< 0.001
*Hydropsyche*	Trichoptera	CF	Clinger	0.24	186	21.6	0.073
Streambed (*n* = 72)							
*Calineuria californica*	Plecoptera	PR	Clinger	0.58	60	51.9	< 0.001
Heptageniida	Ephemeroptera	SC	Clinger	0.51	190	46.2	< 0.001
*Sweltsa*	Plecoptera	PR	Clinger	0.43	39	36.9	< 0.001
*Psephenus*	Coleoptera	SC	Clinger	0.36	72	35.7	< 0.001
*Petrophila*	Lepidoptera	SC	Clinger	0.35	56	33.8	0.002
*Epeorus*	Ephemeroptera	CG	Clinger	0.32	60	31.2	< 0.003
*Hexatoma*	Diptera	PR	Burrower	0.50	20	31.0	0.001
*Sialis*	Megaloptera	PR	Burrower	0.43	18	28.7	0.003
Gomphidae	Odonata	PR	UN	0.38	44	28.5	0.001
*Antocha*	Diptera	CG	Clinger	0.39	36	23.6	0.057
*Acentrella*	Ephemeroptera	CG	Swimmer	0.21	30	19.4	0.018
*Suwallia*	Plecoptera	PR	Clinger	0.17	16	15.9	0.001
*Ferrissia*	Basommatophora	SC	UN	0.11	2	11.1	0.007

*Note:* BFG is behavioral functional group (mode of maintaining position of movement).

### 4.3 Effect of Substrate Type on Aquatic Insect Size

After accounting for aquatic habitat, year and season, there was strong evidence that Diptera (estimate = 0.80 mm; 95% CI = 0.50 mm, 1.09 mm), Plecoptera (estimate = 0.82 mm; 95% CI = 0.53 mm, 1.11 mm), and Coleoptera (estimate = 0.34 mm; 95% CI = 0.23 mm, 0.45 mm) inhabiting streambeds were larger, on average, than organisms from those same orders in bryophytes. In contrast, Odonata (estimate = 2.41 mm; 95% CI = 1.72 mm, 3.41 mm) and Ephemeroptera (estimate = 0.21 mm; 95% CI = 0.11 mm, 0.30 mm) inhabiting bryophytes were larger, on average, compared to streambeds. We found no evidence that substrate type was related to Trichoptera size. After including family as an additional predictor in our models, we found that substrate was no longer related to the size of Plecoptera or Coleoptera (Table S1). However, we still found evidence that Diptera were larger (estimate = 0.71 mm; 95% CI = 0.46 mm, 0.97 mm) in streambeds compared to Diptera in bryophytes and Odonata (estimate = 2.15 mm; 95% CI = 1.09 mm, 3.41 mm) and Ephemeroptera (estimate = 0.12 mm; 95% CI = 0.02 mm, 0.22 mm) were larger in bryophytes compared to streambeds (Table S1).

For Diptera, bryophytes had a slightly higher proportion of smaller taxa, that is, midge and black fly taxa (> 0.92 compared to ~0.89 for streambeds; Table 5). This was particularly the case for the smallest midge taxa observed, Orthocladiinae (mean length = 2.5 mm) which made up 64% of Chironomidae in bryophytes but only 27% in streambeds (Table 5). Bryophytes also had a lower proportion of the largest Dipteran taxa observed (Tipulidae and Ptychopteridae, craneflies; bryophytes ~0.01; streambeds ~0.04) compared to streambeds (Table 5). Cranefly life stage (adult, larva, pupa) did not differ between substrates; larvae made up > 99.7% of craneflies in both substrates. However, the size of cranefly larvae was smaller, on average, in bryophytes (Figure S1), that is, larger taxa (craneflies) as well as larger cranefly larvae from the same taxon contributed higher proportions of Diptera composition in streambeds compared to bryophytes. For Plecoptera, size differences between substrates were related to compositional shifts. Smaller taxa, primarily Neumouridae, accounted for ~68% of Plecoptera in bryophytes but only ~18% in streambeds while larger taxa (e.g., Perlidae) contributed much less to the total make up of Plecoptera in bryophytes compared to streambeds; that is, larger taxa, not larger individuals of the same taxon, made up a much higher percentage of Plecoptera composition in streambeds compared to bryophytes (Table 5). *Calineuria californica* (western stonefly), was one of the most abundant and one of the largest stoneflies, on average, in our study area (Table 5; Figure S2). Western stonefly was also a strong indicator species for streambeds (Table 4) and Perlidae (family containing *Calineuria*) accounted for ~31% of stonefly abundance in streambeds but only ~3% in bryophytes (Table 5). Another large, and less abundant, stonefly *Pteronarcys* (giant salmonfly) was an indicator for bryophytes (Tables 4 and 5; Figure S2). Similar to differentiation in feeding groups between Nemouridae and *C. californica* , *Pteronarcys* like Nemouridae is a shredder whereas *C. californica* is a predator. For Odonata, size differences were driven by larger individuals of the same taxon in bryophytes compared to streambeds, particularly for Coenagrionidae which was abundant in both substrates (Table 5; Figure S2). Ephemeroptera were larger in bryophytes primarily because larger taxa made up a higher percentage of mayfly composition in bryophytes compared to streambeds. The three largest Ephemeroptera families (Ameletidae, Leptophlebiidae, and Ephemerellidae) made up ~70% of mayflies in bryophytes but only ~27% of mayflies in streambeds (Table 5). However, the average length of Heptageniidae (bryophyte = 3.3 mm, streambed = 2.3 mm) was greater in bryophytes compared to streambeds.

**TABLE 5:** Contribution (%) and average length (mm) for dominant invertebrate families from orders (Plecoptera, Coleoptera, Odonata, and Diptera) where average lengths of invertebrates differed between bryophytes and streambeds.

	Bryophyte (%)	Streambed (%)	Length (mm)
Plecoptera			
Chloroperlidae	1	25	3.5
Nemouridae	68	18	2.5
Perlidae	3	31	6
Perlodidae	7	8	3.1
Pteronarcyidae	5	1	5.6
Taeniopterygidae	11	4	3
Other Plecoptera	5	13	3.8
Coleoptera			
Dytiscidae	1	1	3.3
Elmidae	82	67	2.7
Haplidae	1	2	4.1
Hydraenidae	11	1	2.1
Hydrophilidae	3	2	3.3
Psephenidae	< 1	23	3.8
Other Coleoptera	2	4	3
Odonata			
Aeshnidae	8	1	4.1
Coenagrionoidae	80	26	4.2
Gomphidae	9	71	4.7
Other Odonata	3	2	4.5
Ephemeroptera			
Ameletidae	1	2	3.5
Baetidae	18	22	2.3
Ephemerellidae	23	5	2.8
Heptageniidae	1	34	2.3
Leptohypidae	8	15	2.1
Leptophlebiidae	46	20	2.8
Diptera			
Chironomidae	90	87	2.8
Chironominae	*26*	*61*	2.7
Orthocladiinae	*64*	*27*	2.5
*Tanypodinae*	*8*	*10*	3.1
Simuliidae	2	2	2.9
Tipulidae	1	3	7
Ceratopogonidae	7	7	3.9
Ptychopteridae	0	1	17

*Note:* Linear mixed model results testing for the effect of substrate on invertebrate lengths for different orders are reported in Table S1.

## 5 Discussion

### 5.1 Invertebrate Densities and Community Gradients

As hypothesized, bryophytes harbored much greater densities per square meter of aquatic macroinvertebrates compared to streambed substrates. This finding is consistent with numerous studies from temperate streams in Europe (Muotka and Laasonen 2002; Wotton and Merritt 1988), New Zealand (Suren 1991), Japan (Tada and Satake 1994), and North America (Glime 2017; Maurer and Brusven 1983; Munn and Brusven 1991), from an arctic river in Alaska (Lee and Hershey 2000) and from tropical streams in Brazil (Rosa et al. 2011) and Australia (Wulf and Pearson 2017). Invertebrate densities in bryophytes were highest in summer concomitant with the highest invertebrate densities in mid‐order stream reaches and the timing of food limitations for juvenile salmonids in our study system (Kaylor et al. 2020). Summer stream densities of invertebrates in bryophytes averaged between ~60,000 and 70,000 m^−2^ consistent with invertebrate densities reported in stream bryophytes where they are considered “hotspots” of invertebrate productivity (Munn and Brusven 1991; Suren 1991; Wulf and Pearson 2017) as well as woody debris which supports high standing stock densities and biomass of macroinvertebrates in streams (Wallace et al. 1993).

Notably, stream bryophytes can provide “extra” structural habitat for invertebrates above the streambed rather than replace streambed substrates (Janauer and Dokulil 2006). Therefore, the contribution of bryophytes to aquatic invertebrate productivity of a stream reach may be greater than a comparison of invertebrate densities between bryophytes and other benthic substrates would suggest, because they provide productive habitat while also providing “extra” habitat (i.e., structural habitat in different portions of the water column that would otherwise be unstructured). However, bryophyte encroachment can shift invertebrate community composition through the displacement of epilithic algae on rocks and scrapers not adapted to feed in bryophytes (Glime 2017; Lee and Hershey 2000).

Consistent with previous research, bryophytes harbored distinct invertebrate communities compared to other benthic substrates and were dominated by one family of small invertebrates, midges (Glime 2017; Munn and Brusven 1991; Stream Bryophyte Group 1999; Wulf and Pearson 2017). We found that differences in invertebrate composition between bryophytes and streambeds were driven by high dominance of Chironomidae and greater relative abundances of collector‐gatherers and shredders (detritivores) in bryophytes. That primary functional feeding groups responsible for breaking down detritus were most abundant in bryophytes while all other feeding groups were associated with streambeds, provides support for the hypothesis that bryophytes enhance aquatic invertebrate densities because they concentrate food resources for detritivores by trapping organic material (Glime 2017).

Bryophytes are thought to provide food for stream invertebrates primarily through trapping organic matter and supporting periphyton growth on leaf and stem surfaces rather than bryophytes being directly consumed by invertebrates. Direct consumption of bryophytes by aquatic invertebrates is hypothesized to be rare and only common for a few specialized taxa adapted to feed on bryophytes (Glime 2017; Stream Bryophyte Group 1999). However, recent research suggests that consumption of bryophytes, by a wide variety of aquatic invertebrates may be more common than previously hypothesized (Labed‐Veydert et al. 2021). Future research is needed to better understand the direct and indirect paths by which bryophytes provide food resources to invertebrates in different aquatic habitats.

Bryophytes enhanced densities of invertebrates most in summer concomitant with low stream flow conditions. We also found that bryophytes consistently enhanced invertebrate densities compared to streambeds in both wetlands and streams. These results contrast findings from a tropical stream system where invertebrate densities in bryophytes were positively associated with water velocity (Rosa et al. 2011) and provide support against the flow refuge hypothesis as the primary mechanism driving aquatic invertebrate colonization of bryophytes. While we agree with researchers that interactions between bryophytes and small‐scale variations in water velocity influence invertebrate colonization and spatial distributions in stream bryophytes (Glime 2017; Suren 1991; Rosa et al. 2011), our data suggest that this is not the primary factor driving patterns of invertebrate colonization of bryophytes in the Meadow Creek watershed.

Differences between lotic and lentic habitats is considered one of the primary factors influencing aquatic macroinvertebrate distributions (Cummins et al. 2019). Unexpectedly, variation between streams and wetlands was not the primary gradient driving variation in aquatic invertebrate community composition. However, when subdominant taxa were given higher importance (suppressing the weight of dominant taxa such as Chironomidae) in our ordination analysis, differences between streams and wetlands became, by far, the most important factor explaining variation in invertebrate communities (Figure S3). This clearly shows that when Chironomidae were given less weight in defining community structure, the role of bryophytes was primarily to enhance densities of invertebrate communities that were structured by flow dynamics. However, when Chironomidae were allowed to contribute based on their true weight in the community, substrate type—bryophytes versus streambeds—was the main driver of community variation. Therefore, the primary gradient in invertebrate community composition may be best described as a “midge gradient” likely related to greater availability of organic matter trapped in bryophytes compared to streambeds. Our results indicate that regardless of aquatic habitat and season, bryophytes in our study area can be expected to harbor extremely high densities of midges. Our findings combined with research trends around the world including Europe, eastern United States, Alaska, Japan, New Zealand, Australia and Brazil (spanning arctic, temperate, and tropical streams) suggest that riparian bryophytes may be universally considered good habitat for midges; such findings should have major implications for stream and riparian food webs (Averett 2025).

Chironomidae are energetically important insects that provide large contributions of food to many aquatic and terrestrial organisms including aquatic invertebrates (e.g., Ephemeroptera and Plecoptera), arthropods, birds (waterfowl and flycatchers), fish (salmonids and Cyprinids) and even large mammals (e.g., bears; Armitage 1995; Gunther et al. 2018). Chironomidae is the most widespread and one of the most diverse families of aquatic insects and is found in nearly all freshwater habitats from lakes, streams, and wetlands to intermittent streams and even muddy soils (Armitage 1995; Ferrington and Berg 2019). Chironomidae are a highly productive food resource for many organisms globally because they are small, ubiquitous, short‐lived, and among the most tolerant insects to variation in environmental (both physical and chemical) conditions (Armitage 1995). Fluctuations in midge abundance can have major impacts on consumer populations. For example, the productivity of spring midge emergence in northern temperate and arctic regions influences the breeding success of migrating birds (Armitage 1995). Midges (particularly free‐living larvae, e.g., Orthocladiinae) also make up major components of juvenile salmonid diets (Armitage 1995) and are the dominant food resource for rainbow trout and juvenile steelhead ( *Oncorhynchus mykiss* ) in our study area (McLemore 1988).

High tolerance of Chironomidae to environmental conditions makes them dependable food resources (compared to intolerant invertebrates, e.g., Ephemeroptera and Plecoptera) when faced with stressors such as habitat alteration and climate change. Climate change is expected to cause substantial changes to flow regimes (a primary factor influencing aquatic insect distributions; Allan et al. 2021) in the Blue Mountains (Dwire and Mellmann‐Brown 2017). Such changes may result in increased instability (e.g., emergence failure, population declines) of intolerant taxa compared to more tolerant Chironomidae (Pyne and Poff 2017). Therefore, conservation or restoration of habitat that enhances abundance of tolerant aquatic insects such as Chironomidae may boost quantities of a dependable food resource for generalist consumers such as salmonids at a time when increased instability of less tolerant invertebrates may be expected.

While relative abundance patterns showed strong dominance of Chironomidae and detritivores in Bryophytes, absolute abundance patterns suggest high colonization rates of bryophytes by most invertebrate groups. Some researchers have anecdotally suggested that bryophytes may be avoided by larger invertebrate orders that have difficulty navigating small spaces such as Odonata or taxa that are associated with slow‐moving waters such as clams (Veneroida) or aquatic worms (Oligochaeta; Glime 2017). Other research suggests that certain functional feeding groups, such as shredders or predators may avoid bryophytes (Glime 2020; Wulf and Pearson 2017). Our findings do not support general patterns of bryophyte avoidance by specific functional feeding groups or invertebrate orders. Instead, we found that densities of nearly all orders and functional feeding groups were as high—most were higher—in bryophytes compared to streambeds. These patterns were consistent despite variation in water body types and bryophyte composition, and included intolerant taxa associated with flowing waters (e.g., Elmidae, Ephemeroptera, and Plecoptera) as well as more tolerant taxa associated with slow to still water habitats (e.g., Veneroida, Odonata, Amphipoda and Oligochaeta). Our results revealed negative associations between relative abundances for all feeding functional groups except for collector‐gathers and shredders with bryophytes. However, those negative relationships were primarily driven by disproportional enhancement of collector‐gathers and shredders in bryophytes rather than decreased absolute abundances of other feeding groups (e.g., predators and scrapers).

While most orders and functional feeding groups were more abundant in bryophytes, at a finer taxonomic level (sub‐order), we found evidence that specific taxa—primarily predators and scrapers—showed moderate to strong affinities towards streambeds. For example, Ephemeroptera were more abundant in bryophytes; however, scrapers such as Heptageniidae, had tendencies towards streambeds. Whereas other Ephemeroptera, primarily collector‐gatherers, showed tendencies towards bryophytes. Similarly, for Plecoptera, collector‐gatherers and shredders, for example, Neumouridae and *Pteronarcys*, were indicators of bryophytes, while predators, for example, *C. californica* , had affinities for streambeds. Such patterns align with hypotheses that some aquatic macroinvertebrate species segregate between bryophytes and streambeds based on feeding strategies. Contrasting tendencies for specific taxa towards bryophytes versus streambeds, presumably influenced by feeding strategy, resulted in increased community dissimilarity and are in accordance with Cummins et al. (2019), that aquatic invertebrate diversity is positively associated with aquatic substrate heterogeneity.

### 5.2 Effect of Substrate Type on Aquatic Insect Size

We found evidence that for Diptera, Plecoptera, and Coleoptera, invertebrates were larger, on average, in streambeds compared to bryophytes. Previous research proposed that bryophytes may serve as nurseries for some larger aquatic insects where early instars of taxa such as Plecoptera inhabit bryophytes while the larger, later instars move to streambed substrates (Glime 2020; Suren 1991; Wulf and Pearson 2017). For Coleoptera, Plecoptera, and Trichoptera, we found no evidence that invertebrate size differed between substrate types after accounting for invertebrate family. However, we did find evidence for a subset of “larger” taxa including several cranefly taxa (Diptera), that larvae were substantially larger in streambeds compared to bryophytes. Our results suggest that larger Diptera, Plecoptera, and Coleoptera in streambeds compared to bryophytes were driven primarily by compositional differences rather than a “nursery‐effect”, that is, for the most part, smaller taxa made up higher proportions of the invertebrate composition in bryophytes compared to streambeds rather than smaller individuals of the same taxon segregating by substrate type. Western stonefly was one of the largest and most abundant stoneflies in our dataset. The strong affinity of the western stonefly for streambeds was the primary reason why Plecoptera were larger in streambeds compared to bryophytes. Our finding that *Pteronarcys* (giant salmonfly), a similarly large stonefly as the western stonefly, was an indicator of bryophytes suggests that taxa such as the western stonefly may not be excluded from bryophytes because they are too large to navigate them. Instead, our results suggest that affinities for giant salmonfly, a detrital shredder, towards bryophytes and western stonefly, a predator, towards streambeds were likely related to differences in feeding strategies. It is unclear whether survival rates for small instars of crane flies are higher in bryophytes and/or if larger instars of these taxa preferentially select streambeds as habitat. Rare observations of large craneflies in bryophytes suggest the latter. Future research will be needed to better understand how life stage and size influence colonization of bryophytes by specific taxa such as craneflies, Odonata, and Plecoptera.

### 5.3 Management Implications

Stream restoration seeks to restore natural processes that create and maintain high‐quality habitat (Roni et al. 2002) supporting both the physical habitat and food requirements for threatened stream biota (Wipfli and Baxter 2010). Bryophytes play an integral role in these processes that are the focus of stream restoration. Our results demonstrate that riparian bryophytes are productive habitat for most macroinvertebrate functional groups across headwater and mid‐order streams and wetlands in an interior PNW salmonid‐bearing stream system. Bryophytes were particularly productive habitat for midges, a highly productive, tolerant, and energetically important insect family. However, bryophytes are currently an undervalued and understudied vegetation group that have generally not been considered in stream‐riparian restoration in the PNW. Our findings illustrate that bryophytes provide an important ecological role as habitat for diverse and productive riparian invertebrate communities in interior PNW streams and floodplains and provide support for their explicit consideration in stream and riparian restoration planning and management to support highly productive aquatic invertebrate communities and predators that rely on them.

## Author Contributions

**Joshua P. Averett:** conceptualization (lead), data curation (lead), formal analysis (lead), investigation (lead), methodology (lead), supervision (supporting), validation (lead), visualization (lead), writing – original draft (lead), writing – review and editing (lead). **Leslie M. Naylor:** conceptualization (supporting), funding acquisition (equal), investigation (supporting), methodology (supporting), project administration (equal), resources (equal), writing – review and editing (supporting). **David E. Wooster:** conceptualization (supporting), investigation (supporting), methodology (supporting), resources (supporting), writing – review and editing (supporting). **Amanda Hardman:** investigation (supporting), methodology (supporting), writing – review and editing (supporting). **Michael J. Wisdom:** conceptualization (supporting), funding acquisition (equal), investigation (supporting), project administration (equal), writing – review and editing (supporting). **Bryan A. Endress:** conceptualization (supporting), funding acquisition (equal), investigation (supporting), methodology (supporting), project administration (equal), resources (equal), supervision (lead), writing – review and editing (supporting).

## Funding

This work was supported by the Confederated Tribes of the Umatilla Indian Reservation, Bureau of Reclamation, Oak Ridge Institute for Science and Education, Oregon State University, and US Forest Service Pacific Northwest Research Lab.

## Conflicts of Interest

The authors declare no conflicts of interest.

## Supporting information


**Appendix S1:** ece372627‐sup‐0001‐AppendixS1.docx.

## Data Availability

Data used in this study is provided publicly to use for further analyses and to replicate our findings. Raw data and metadata are accessible through ScholarsArchive@OSU, DOI: https://doi.org/10.7267/f1881v99f.
