# Supplementary material for: Riparian Bryophytes: An Overlooked Yet Important Habitat for Aquatic Macroinvertebrates in Interior Pacific Northwest (USA) Salmonid‐Bearing Streams
Source: Ecol Evol. 2025 Dec 16;15(12):e72627. doi: 10.1002/ece3.72627 (PMC12706411; doi:10.1002/ece3.72627)
Supplement: Supplementary file 1 — Appendix S1: ece372627‐sup‐0001‐AppendixS1.docx. [file ECE3-15-e72627-s001.docx]

APPENDIX S1

Table S1: Linear mixed model regression results showing the fixed effects of predictors (e.g., substrate) on invertebrate length for dominant aquatic insect orders. Significance codes: 0 ‘***’ 0.001 ‘**’ 0.01 ‘*’ 0.05 ‘.’ 0.1 ‘ ’ 1.

| **Diptera** | | | | | | |
| --- | --- | --- | --- | --- | --- | --- |
|  | Estimate | Std. Error | df | t value | Pr(>\|t\|) |  |
| (Intercept) | 853.4832 | 317.6896 | 2264.2943 | 2.687 | 0.007273 | ** |
| substrateStreambed | 0.7113 | 0.1316 | 2631.8558 | 5.405 | 7.05E-08 | *** |
| habitatMidOrder | 0.1284 | 0.3141 | 9.4326 | 0.409 | 0.691892 |  |
| habitatWetland | 0.5977 | 0.3605 | 9.2329 | 1.658 | 0.130811 |  |
| year2023 | -0.4189 | 0.1571 | 2262.595 | -2.666 | 0.007723 | ** |
| seasonSPRING | 0.3093 | 0.1927 | 2507.1266 | 1.606 | 0.1085 |  |
| seasonSUMMER | -0.3386 | 0.1498 | 2771.6959 | -2.261 | 0.023863 | * |
| familyCeratopogonidae | -2.6914 | 0.9244 | 2841.7461 | -2.912 | 0.003623 | ** |
| familyChironomidae | -3.8128 | 0.9109 | 2850.5915 | -4.186 | 2.93E-05 | *** |
| familyOtherDiptera | -4.6204 | 1.5603 | 2868.2445 | -2.961 | 0.003089 | ** |
| familyDixidae | -2.1212 | 1.1763 | 2866.6832 | -1.803 | 0.071465 | . |
| familyDolichopodidae | -1.8536 | 2.5312 | 2860.6893 | -0.732 | 0.464049 |  |
| familyEmpididae | -4.7544 | 1.2011 | 2868.8991 | -3.958 | 7.74E-05 | *** |
| familyEphydridae | -4.8051 | 2.1439 | 2867.7826 | -2.241 | 0.025081 | * |
| familyMuscidae | 1.2768 | 1.7486 | 2866.8845 | 0.73 | 0.465326 |  |
| familyPsychodidae | -3.3827 | 1.3964 | 2869 | -2.422 | 0.015481 | * |
| familyPtychopteridae | 12.267 | 1.0835 | 2861.9202 | 11.322 | < 2e-16 | *** |
| familySciomyzidae | -4.5429 | 3.4742 | 2865.5115 | -1.308 | 0.191112 |  |
| familySimuliidae | -3.6623 | 0.9562 | 2851.9434 | -3.83 | 0.000131 | *** |
| familyStratiomyidae | 0.6195 | 1.2071 | 2868.5694 | 0.513 | 0.607873 |  |
| familySyrphidae | 0.5083 | 1.7578 | 2868.9799 | 0.289 | 0.772473 |  |
| familyTabanidae | 1.7473 | 1.1507 | 2866.413 | 1.518 | 0.129014 |  |
| familyTipulidae | 0.5433 | 0.9281 | 2862.2518 | 0.585 | 0.558297 |  |
| **Plecoptera** | | | | | | |
| (Intercept) | 1.72356 | 0.58305 | 38.87936 | 2.956 | 0.00527 | ** |
| substrateStreambed | -0.04948 | 0.17057 | 1305.4987 | -0.29 | 0.77179 |  |
| habitatMidOrder | 0.25991 | 0.46687 | 7.24754 | 0.557 | 0.5945 |  |
| habitatWetland | 0.74124 | 0.94354 | 17.00666 | 0.786 | 0.44291 |  |
| year2023 | -0.11517 | 0.16625 | 1225.1461 | -0.693 | 0.4886 |  |
| seasonSPRING | -0.02629 | 0.22042 | 1312.1106 | -0.119 | 0.90509 |  |
| seasonSUMMER | -0.99459 | 0.15489 | 1293.4836 | -6.421 | 1.89E-10 | *** |
| familyChloroperlidae | 2.29256 | 0.47725 | 1313.8459 | 4.804 | 1.74E-06 | *** |
| familyLeuctridae | 3.68866 | 1.61425 | 1310.7983 | 2.285 | 0.02247 | * |
| familyNemouridae | 1.18976 | 0.45726 | 1311.0566 | 2.602 | 0.00937 | ** |
| familyPeltoperlidae | 0.81261 | 1.61966 | 1309.9974 | 0.502 | 0.61595 |  |
| familyPerlidae | 3.907 | 0.47078 | 1313.8156 | 8.299 | 2.58E-16 | *** |
| familyPerlodidae | 1.91169 | 0.48444 | 1313.6676 | 3.946 | 8.36E-05 | *** |
| familyOtherPlecoptera | -0.50989 | 0.55919 | 1303.7736 | -0.912 | 0.36203 |  |
| familyPteronarcyidae | 5.32411 | 0.61986 | 1309.5047 | 8.589 | < 2e-16 | *** |
| familyTaeniopterygidae | 1.03425 | 0.53798 | 1281.2053 | 1.922 | 0.05477 | . |

Table S1 Continued: Linear mixed model regression results showing the fixed effects of predictors (e.g., substrate) on invertebrate length for dominant aquatic insect orders. Significance codes: 0 ‘***’ 0.001 ‘**’ 0.01 ‘*’ 0.05 ‘.’ 0.1 ‘ ’ 1.

| **Coleoptera** | | | | | | |
| --- | --- | --- | --- | --- | --- | --- |
|  | Estimate | Std. Error | df | t value | Pr(>\|t\|) |  |
| (Intercept) | 3.8217 | 0.43154 | 103.40418 | 8.856 | 2.49E-14 | *** |
| substrateStreambed | 0.11149 | 0.05932 | 2225.35851 | 1.88 | 0.060304 | . |
| habitatMidOrder | 0.0339 | 0.26631 | 7.24854 | 0.127 | 9.02E-01 |  |
| habitatWetland | -0.0598 | 0.34468 | 11.13436 | -0.173 | 0.865378 |  |
| year2023 | -0.43879 | 0.06519 | 2207.74638 | -6.731 | 2.15E-11 | *** |
| seasonSPRING | 0.06014 | 0.08391 | 2231.275 | 0.717 | 0.473635 |  |
| seasonSUMMER | -0.03096 | 0.05795 | 2229.65396 | -0.534 | 0.593161 |  |
| familyChrysomelidae | 1.50393 | 0.42595 | 2171.95666 | 3.531 | 0.000423 | *** |
| familyOtherColeoptera | 5.82038 | 1.24225 | 2226.28041 | 4.685 | 2.96E-06 | *** |
| familyCurculionidae | -1.21893 | 0.91923 | 2227.62063 | -1.326 | 1.85E-01 |  |
| familyDytiscidae | -0.53664 | 0.41275 | 2215.9969 | -1.300 | 0.19368 |  |
| familyElmidae | -1.05499 | 0.37198 | 2199.16902 | -2.836 | 0.004608 | ** |
| familyHaliplidae | 0.14803 | 0.40776 | 2173.66286 | 0.363 | 0.716626 |  |
| familyHydraenidae | -1.5899 | 0.3761 | 2205.82992 | -4.227 | 2.46E-05 | *** |
| familyHydrophilidae | -0.48177 | 0.38944 | 2179.17414 | -1.237 | 0.216194 |  |
| familyLampyridae | 1.25077 | 1.24841 | 2230.52649 | 1.002 | 0.316505 |  |
| familyPsephenidae | -0.37761 | 0.38425 | 2208.27074 | -0.983 | 0.325847 |  |
| familyScirtidae | -0.81595 | 0.49975 | 2185.82255 | -1.633 | 0.102674 |  |
| familyStaphylinidae | 0.87289 | 0.44346 | 2229.95009 | 1.968 | 0.049151 | * |
| **Ephemeroptera** | | | | | | |
| (Intercept) | 3.82842 | 0.20532 | 14.51058 | 18.646 | 1.53E-11 | *** |
| substrateStreambed | -0.12137 | 0.0515 | 3195.24072 | -2.357 | 0.0185 | * |
| habitatMidOrder | -0.09848 | 0.18205 | 3.99189 | -0.541 | 0.6173 |  |
| habitatWetland | 1.87471 | 0.33785 | 21.22162 | 5.549 | 1.60E-05 | *** |
| year2023 | -0.28767 | 0.06479 | 3082.52267 | -4.44 | 9.32E-06 | *** |
| seasonSPRING | 0.02176 | 0.06957 | 3170.45119 | 0.313 | 0.75452 |  |
| seasonSUMMER | -0.26694 | 0.06082 | 3139.59693 | -4.389 | 1.18E-05 | *** |
| familyBaetidae | -1.19254 | 0.1454 | 3204.70987 | -8.202 | 3.39E-16 | *** |
| familyCaenidae | -1.33731 | 0.19163 | 3213.40117 | -6.979 | 3.60E-12 | *** |
| familyEphemerellidae | -0.72514 | 0.14774 | 3211.00575 | -4.908 | 9.65E-07 | *** |
| familyOtherEphemeroptera | -0.97430 | 0.34317 | 3207.27646 | -2.839 | 0.00455 | ** |
| familyHeptageniidae | -1.07907 | 0.14699 | 3205.72134 | -7.341 | 2.67E-13 | *** |
| familyLeptohyphidae | -1.42896 | 0.15368 | 3206.12555 | -9.298 | < 2e-16 | *** |
| familyLeptophlebiidae | -0.62933 | 0.14641 | 3204.47611 | -4.298 | 1.77E-05 | *** |

Table S1 Continued: Linear mixed model regression results showing the fixed effects of predictors (e.g., substrate) on invertebrate length for dominant aquatic insect orders. Significance codes: 0 ‘***’ 0.001 ‘**’ 0.01 ‘*’ 0.05 ‘.’ 0.1 ‘ ’ 1.

| **Trichoptera** | | | | | | |
| --- | --- | --- | --- | --- | --- | --- |
|  | Estimate | Std. Error | df | t value | Pr(>\|t\|) |  |
| (Intercept) | 2.07989 | 0.26673 | 14.03478 | 7.798 | 1.81E-06 | *** |
| substrateStreambed | 0.11921 | 0.13564 | 1197.87556 | 0.879 | 0.3797 |  |
| habitatMidOrder | -0.30659 | 0.29712 | 10.02976 | -1.032 | 0.3264 |  |
| habitatWetland | -1.24835 | 0.86889 | 335.05877 | -1.437 | 0.1517 |  |
| year2023 | -0.08986 | 0.14702 | 856.56255 | -0.611 | 0.5412 |  |
| seasonSPRING | 1.80299 | 0.23459 | 1240.15685 | 7.686 | 3.08E-14 | *** |
| seasonSUMMER | -0.73955 | 0.15879 | 1046.96532 | -4.657 | 3.62E-06 | *** |
| familyGlossosomatidae | 0.09212 | 1.63128 | 1267.21432 | 0.056 | 0.955 |  |
| familyHelicopsychidae | 0.49041 | 0.77792 | 1085.43282 | 0.63 | 0.5286 |  |
| familyHydropsychidae | 2.13684 | 0.24501 | 1189.40252 | 8.721 | < 2e-16 | *** |
| familyHydroptilidae | 0.69582 | 0.28393 | 1269.12335 | 2.451 | 0.0144 | * |
| familyLepidostomatidae | 1.29919 | 0.21731 | 1228.07385 | 5.978 | 2.95E-09 | *** |
| familyLimnephilidae | 3.12282 | 0.35084 | 1259.76777 | 8.901 | < 2e-16 | *** |
| familyPhilopotamidae | 1.50221 | 0.31961 | 1066.11299 | 4.7 | 2.94E-06 | *** |
| familyPolycentropodidae | 2.50127 | 0.45213 | 1283.88901 | 5.532 | 3.83E-08 | *** |
| familyPsychomyiidae | 3.70251 | 0.88576 | 1256.41164 | 4.18 | 3.12E-05 | *** |
| familyRhyacophilidae | 3.25216 | 0.47135 | 1198.17499 | 6.9 | 8.42E-12 | *** |
| familyOtherTrichoptera | -0.31415 | 1.61793 | 1284.62052 | -0.194 | 0.846 |  |
| familyUenoidae | 1.04608 | 1.03965 | 1282.89544 | 1.006 | 0.3145 |  |
| **Odonata** | | | | | | |
| (Intercept) | 5.78973 | 1.69803 | 98.98455 | 3.41 | 9.42E-04 | *** |
| substrateStreambed | -2.15103 | 0.61047 | 97.40079 | -3.524 | 0.00065 | *** |
| habitatMidOrder | 3.66534 | 2.22189 | 138.79025 | 1.65 | 0.10128 |  |
| habitatWetland | 1.91763 | 2.32807 | 124.57305 | 0.824 | 0.41169 |  |
| year2023 | -1.5934 | 0.4769 | 35.72874 | -3.341 | 0.00196 | ** |
| seasonSPRING | 0.27406 | 0.83121 | 165.57321 | 0.33 | 7.42E-01 |  |
| seasonSUMMER | -0.04941 | 0.45977 | 89.88592 | -0.107 | 9.15E-01 |  |
| familyCalopterygidae | 3.20205 | 3.85204 | 294.37358 | 0.831 | 0.4065 |  |
| familyCoenagrionidae | -3.05883 | 1.44254 | 298.99993 | -2.12 | 0.03479 | * |
| familyCordulegastridae | 2.02551 | 3.94417 | 297.73505 | 0.514 | 0.60795 |  |
| familyGomphidae | -3.29453 | 1.49288 | 287.40969 | -2.207 | 0.02812 | * |
| familyLibellulidae | 0.19525 | 2.00734 | 298.03068 | 0.097 | 9.23E-01 |  |


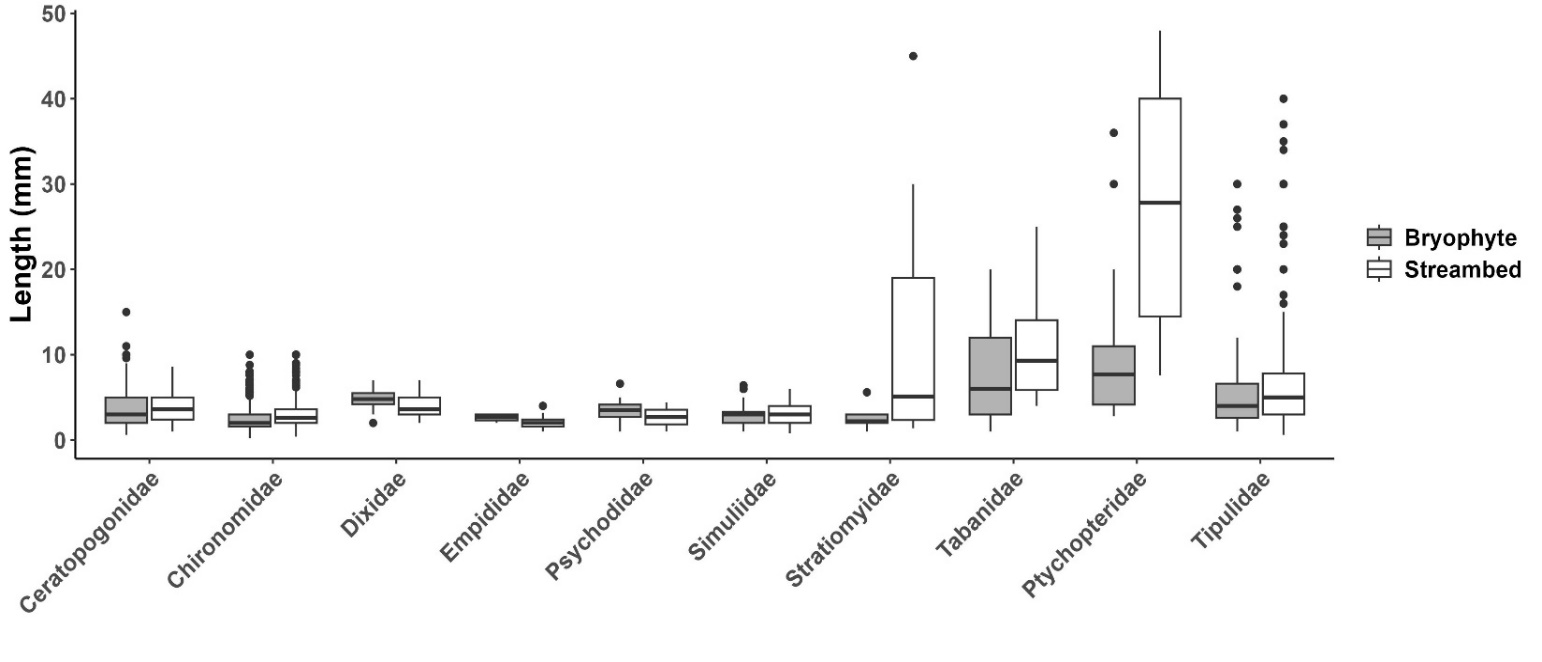

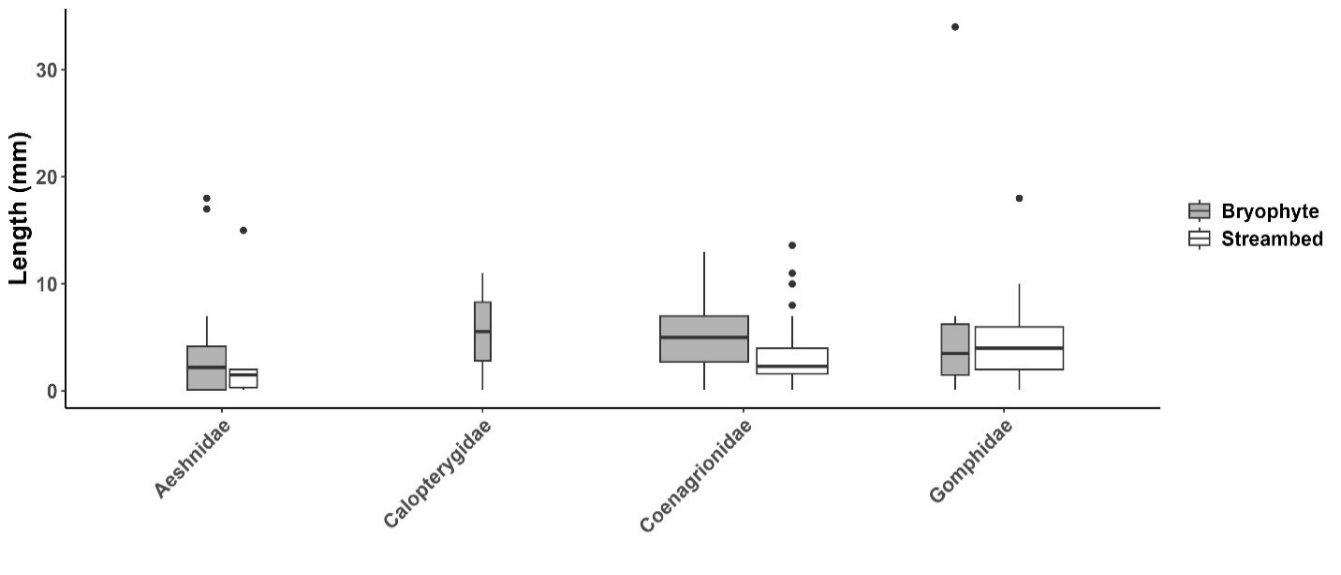


**Diptera**

Figure S1: Boxplots for Diptera length measurements stratified by family and substrate type across aquatic habitats (headwaters, mid-order streams, and floodplain wetlands).


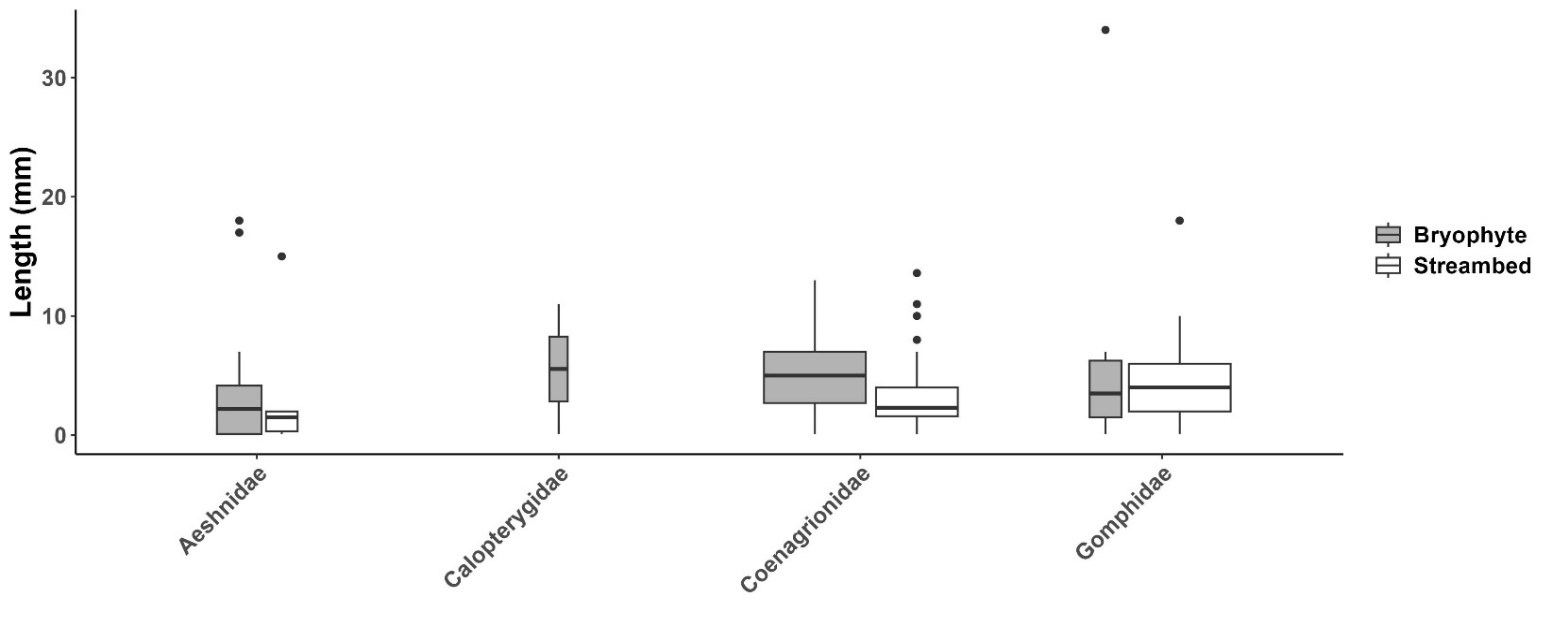


**Odonata**


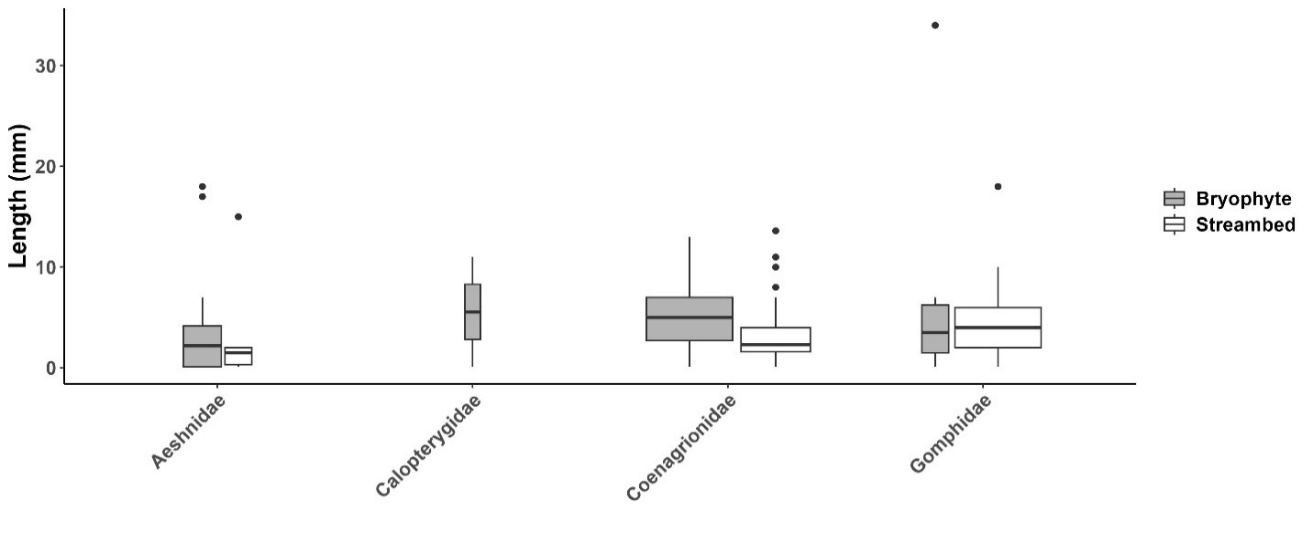

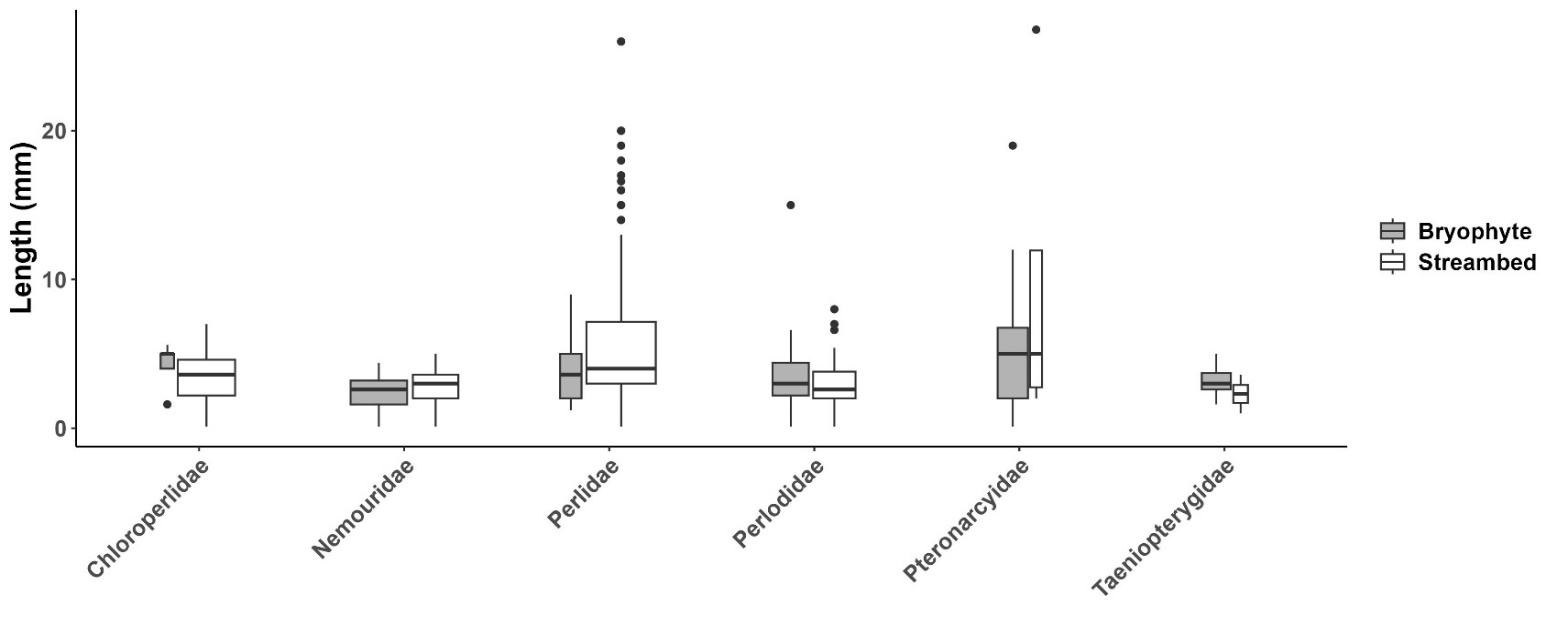


**Plecoptera**


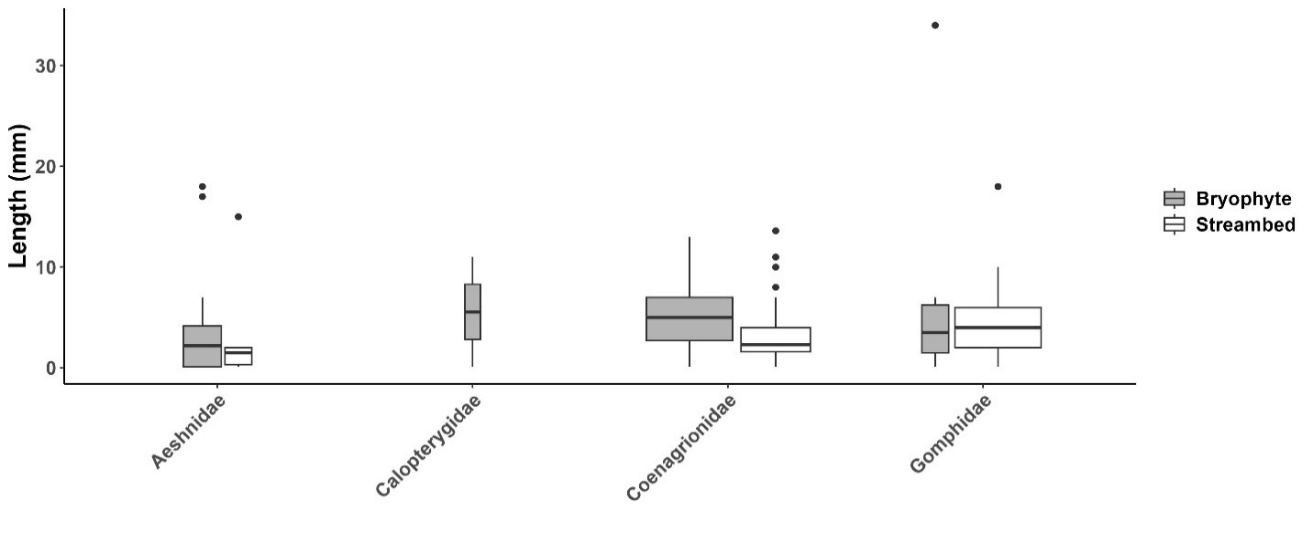


Figure S2: Boxplots for Plecoptera (top) and Odonata (bottom) length measurements stratified by family and substrate type across aquatic habitats (headwaters, mid-order streams, and floodplain wetlands). Width of boxplots are proportional to sample size.


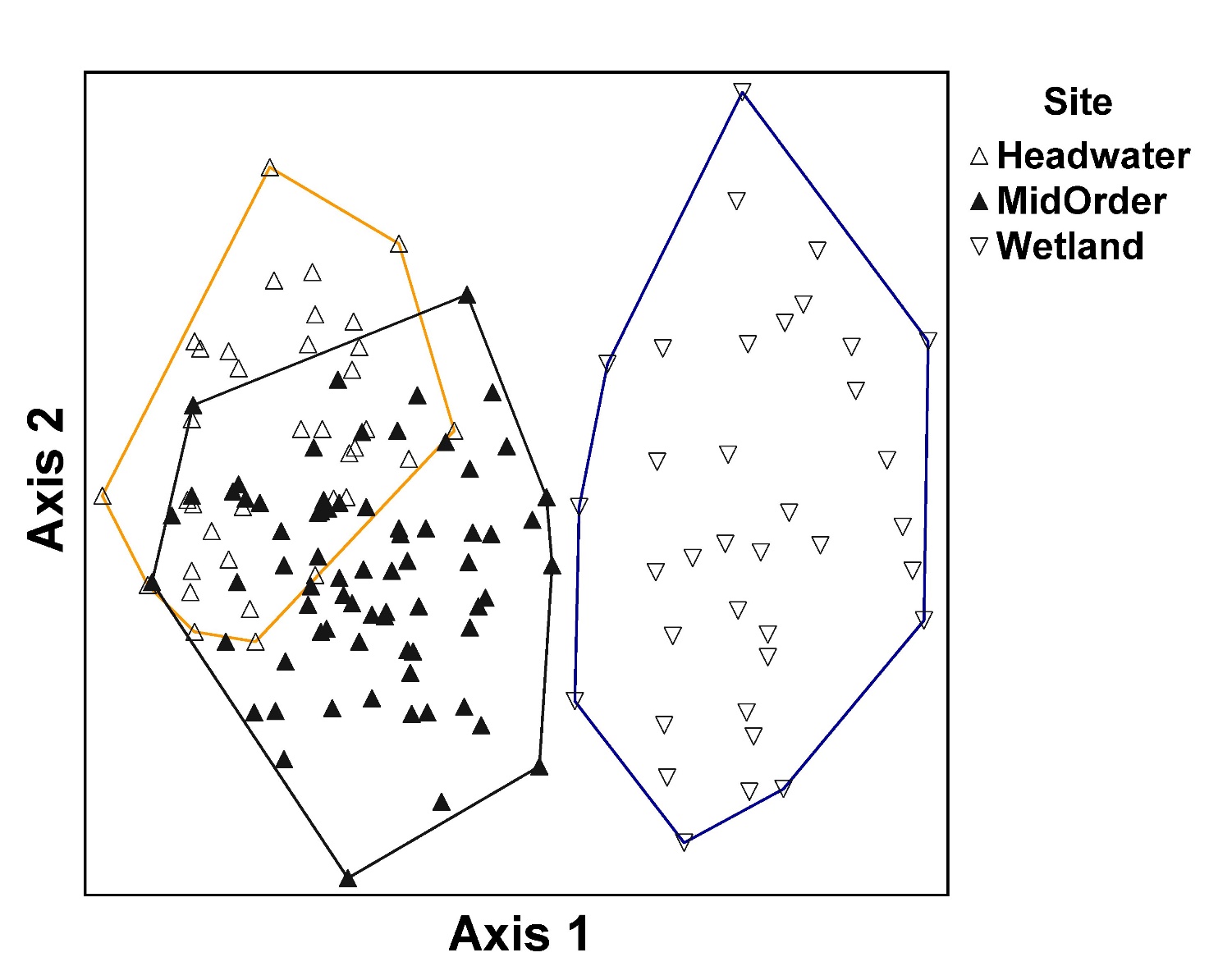


Figure S3: Non-metric multidimensional scaling ordination (NMS) plot of aquatic habitat sample units in macroinvertebrate community space (order-level). Invertebrate abundances for this plot were log-transformed using a generalized log transformation, b = log(x+xmin) – log(xmin), where xmin was the smallest nonzero value in the matrix, because the smallest non-zero numbers were much less than one. NMS was performed using a Sørenson distance measure, Kruskal’s strategy 1 for penalization of ties, a random starting configuration with maximum of 500 iterations, and the slow and thorough autopilot setting. Transformation of abundances, i.e., giving dominant species (e.g., Chironomidae) less weight in defining the ordination space flipped Axes 1 and Axes 2, i.e., now flow dynamics (lentic *vs.* lotic) drive community variation. Axis 1 explained 63% of variation in the distance matrix; Axis 2, which was still strongly associated with separation of bryophytes from the stream bed explained 16% of variation in the distance matrix.
